# Computational Analysis of *CC2D1A* Missense Mutations: Insight into Protein Structure and Interaction Dynamics

**DOI:** 10.1021/acschemneuro.4c00570

**Published:** 2025-01-10

**Authors:** Anwar Abuelrub, Ismail Erol, Nurdeniz Nalbant Bingol, Sebnem Ozemri Sag, Sehime G. Temel, Serdar Durdağı

**Affiliations:** † Laboratory for Innovative Drugs (Lab4IND), Computational Drug Design Center (HITMER), Bahçeşehir University, 34734 İstanbul, Türkiye; ‡ Computational Biology and Molecular Simulations Laboratory, Department of Biophysics, School of Medicine, Bahçeşehir University, 34734 Istanbul, Türkiye; § Graduate School of Natural and Applied Sciences, Artificial Intelligence Program, Bahçeşehir University, 34734 Istanbul, Turkey; ∥ Department of Analytical Chemistry, School of Pharmacy, Bahçeşehir University, 34351 İstanbul, Türkiye; ⊥ Department of Translational Medicine, Institute of Health Sciences, Bursa Uludag University, 16059 Bursa, Türkiye; # Department of Medical Genetics, Faculty of Medicine, Bursa Uludag University, 16059 Bursa, Türkiye; ∇ Department of Histology and Embryology, Faculty of Medicine, Bursa Uludag University, 16059 Bursa, Türkiye; ○ Molecular Therapy Laboratory, Department of Pharmaceutical Chemistry, School of Pharmacy, Bahçeşehir University, 34351 İstanbul, Türkiye

**Keywords:** *CC2D1A*, molecular simulations, neighborhood interaction analyses, autism

## Abstract

*CC2D1A* is implicated in a range of conditions, including autism spectrum disorder, intellectual disability, seizures, autosomal recessive nonsyndromic intellectual disability, heterotaxy, and ciliary dysfunction. In order to understand the molecular mechanisms underlying these conditions, we focused on the structural and dynamic activity consequences of mutations within this gene. In this study, whole exome sequencing identified the c.1552G > A (GLU518LYS) missense mutation in the *CC2D1A* in an 18-year-old male, linking it to intellectual disability and autism. In addition to the GLU518LYS mutation, we conducted a comprehensive analysis of other predefined missense mutations (i.e., PRO192LEU, GLN506ARG, PRO532LEU, GLY781VAL, and GLY781GLU) found within the *CC2D1A*. Utilizing all-atom molecular dynamics (MD) simulations and neighborhood interaction analyses, we delve into the impact of these mutations on protein structure and function at an atomic level, aiming to shed light on their contribution to the pathogenesis of related diseases. The results suggest that GLU518LYS, GLY781VAL, and GLY781GLU mutations did not significantly alter overall global protein structure compared to the wild type, while PRO192LEU, GLN506ARG, and PRO532LEU exhibited slightly higher protein root-mean-square deviation (RMSD) values, which may indicate potential impacts on whole protein stability. Moreover, neighborhood interaction analysis indicated that ASP85 emerges as a unique interaction partner specifically associated with the GLU518LYS mutation, whereas LYS75, which interacts with the ASP85 in the mutated form, is absent in the wild type. This alteration signifies a crucial reconfiguration in the local interaction network at the site of the mutation.

## Graphical abstract

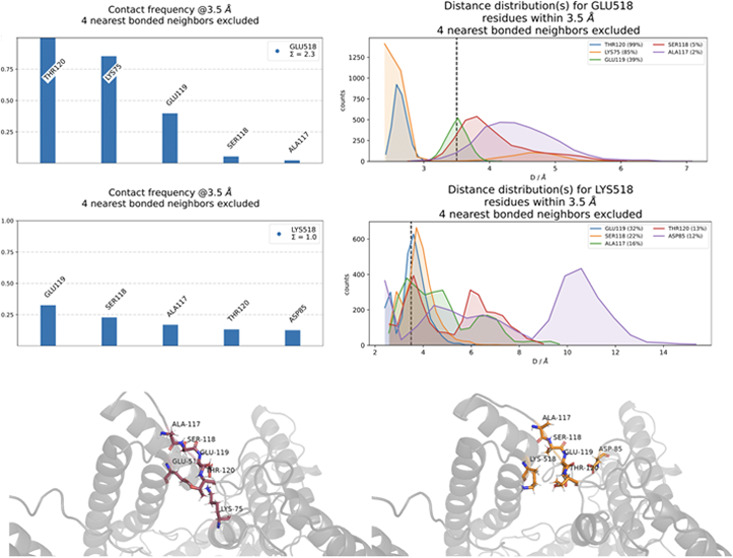

## Introduction

The Coiled-Coil and C2 Domain Containing 1A (*CC2D1A*), also known as *MRT3*, is essential in human biology, particularly in neurological development and function.^1^ Mutations or dysregulation of this gene have been associated with various medical conditions, emphasizing its significance in human health.^71–6^ *CC2D1A*, located on chromosome 19p13.12, encodes protein spans 950 amino acid residues and has a molecular weight of approximately 104 kDa.^1,2^ It is an evolutionarily conserved protein comprising four DM14 domains at its N-terminus and a C2 domain at its C-terminus.^8^ Its structural features suggest involvement in protein–protein interactions and membrane binding, respectively.^8^ It interacts with multiple proteins involved in these processes, such as Rab proteins and the phosphoinositide 3-kinase (PI3K) pathway components, suggesting its role in coordinating intracellular signaling and membrane dynamics.^9^ The CC2D1A protein is functionally implicated in several cellular processes critical to neurodevelopment. These include its role in transcription factor nuclear factor-κB (NF-κB),^8,10^ the regulation of dopamine D2 receptors and 5-hydroxytriptamine serotonin 1A (5-HT1A),^11,11^ as well as its involvement in key signaling pathways such as bone morphogenetic protein (BMP) signaling^13^ and Notch signaling.^14^ Moreover, alternative splicing of the *CC2D1A* mRNA leads to the generation of multiple isoforms with potentially distinct functions.^15^ *CC2D1A* is predominantly expressed in the brain, particularly during embryonic development, indicating its importance in neurodevelopmental processes.^16^ Furthermore, studies have suggested potential links between *CC2D1A* and other neurological conditions, such as intellectual disability (ID), recessive nonsyndromic intellectual disability (NSID), autism spectrum disorder (ASD), seizures, schizophrenia, and autosomal recessive intellectual disability (ARID).^3,4,6,12,17^ Although the exact mechanisms remain to be elucidated, dysregulation of *CC2D1A*-mediated pathways could contribute to the pathogenesis of these disorders.^8^ Our recent study highlighted that *CC2D1A* is pivotal not only in intellectual disabilities but also in ciliopathies, heterotaxy, and renal dysplasia.^2^ Additionally, we provide functional validation for novel variants linked to this emerging condition and offer new insights into the role of *CC2D1A* in ciliary biology. These results underscore the critical role of *CC2D1A* in developmental processes and its link to various congenital disorders.

Previous studies have identified a strong link between *CC2D1A* mutations and various human diseases. Such as, a notable study by Ma et al.^18^ extensively investigates the role of monoallelic mutations in *CC2D1A*, uncovering its novel contribution to human heterotaxy and ciliary dysfunction. Through a comprehensive analysis of patient samples, the study identifies several mutations in *CC2D1A*, including c.575C > T, resulting in the amino acid mutation p. (PRO192LEU); c.1517A > G, leading to p. (GLN506ARG); c.1595C > T, resulting in p. (PRO532LEU); c.2342G > T, causes p. (GLY781VAL); and c.2342G > A, leading to p. (GLY781GLU). These mutations impact critical amino acid residues within the protein’s DM14 domains and C2 domain, regions essential for its structural integrity and functional roles in ciliary biology. The study highlights how these alterations contribute to heterotaxy syndrome and ciliary abnormalities, providing essential insights into the molecular mechanisms underlying these conditions. Despite these findings, the broader implications of *CC2D1A* dysfunction in multisystem disorders remain poorly understood. In particular, understanding how missense mutations disrupt protein structure and function presents significant challenges. Subtle amino acid substitutions can destabilize protein folding, affect functional domains, or interfere with key interactions, such as those involving catalytic residues or ligand-binding sites.^19^ Compared to large-scale genomic alterations, the impact of missense mutations is less predictable, requiring extensive experimental validation, which is both costly and time-intensive. To bridge this knowledge gap, our study employs all-atom molecular dynamics (MD) simulations, a powerful computational tool for exploring the structural and dynamic consequences of missense mutations at the molecular level. By simulating the behavior of *CC2D1A* variants in physiologically relevant conditions, MD simulations provide valuable insights into how these mutations impact protein stability, folding pathways, and functional interactions. This approach enables a deeper understanding of the molecular basis of *CC2D1A*-associated disorders, facilitating the identification of mechanisms underlying disease phenotypes and informing future therapeutic strategies.^19–22^

In this study, we aim to analyze the microenvironmental interactions of the c.1552G > A (GLU518LYS) missense mutation in the *CC2D1A*, identified in an 18-year-old male patient with intellectual disability and ASD. Additionally, we investigated previously reported mutations by Ma et al.,^18^ which have been pivotal in shedding light on the complex role of CC2D1A in human autism, heterotaxy, and ciliary dysfunction. This work is intended to contribute to future discoveries by providing a deeper understanding of the molecular mechanisms underlying the effects of *CC2D1A* mutations. By elucidating these mechanisms, our study aims to inform the development of targeted therapeutic strategies that could potentially mitigate the impact of such mutations on cognitive and developmental disorders, particularly those associated with ASD, ID, and other related conditions.

## Methods

### DNA Extraction

Genomic DNA was extracted from peripheral venous blood using the QIAamp DNA Mini Kit (QIAGEN, Ankara, Türkiye).

### Whole Exome Sequencing (WES)

For whole exome sequencing (WES), the SureSelectXT Library Prep Kit was utilized for target enrichment of the protein-coding regions of the genome. All procedures were carried out according to the manufacturer’s protocols. Paired-end sequencing was performed on an Illumina NovaSeq system with a read length of 151 base pairs. Base calling and image analysis were conducted using Illumina’s Real-Time Analysis software. The BCL (base calls) binary file generated by the sequencing instrument was then converted into FASTQ format, which contains the sequence data and corresponding quality scores, utilizing Illumina’s bcl2fastq package.

### Bioinformatics Analysis

All bioinformatics analysis was performed on the Sophia DDMTM platform, which includes algorithms for alignment, calling SNPs and small indels (Pepper), calling copy number variations (Muskat), and functional annotation (Moka). Raw reads were aligned to the human reference genome (GRCh37/hg19). Variant filtering and interpretation were performed on Sophia DDMTM. Integrative Genomics Viewer (IGV)^23^ was used to bam file visualization. In families with consanguineous marriages, homozygosity mapping was carried out with HomSI.^24^

### *CC2D1A:* Variant Selection and Classification

To investigate potential pathogenic variants in the *CC2D1A*, we analyzed the genetic sequencing data of an 18-year-old male patient diagnosed with autism. In addition to five missense variants in heterotaxy were identified as candidates for further analysis: c.575C > T (PRO192LEU), c.1517A > G (GLN506ARG), c.1595C > T (PRO532LEU), c.2342G > T (GLY781VAL), and c.2342G > A (GLY781GLU). The classification of these variants was performed using multiple bioinformatics tools and databases, including Franklin, Varsome, the Human Genomics Community, and the Human Gene Mutation Database (HGMD).^25–27^ These resources provided information regarding the potential pathogenicity and clinical relevance of the identified variants. To further assess the functional impact of each variant, an *in silico* analysis was performed utilizing multiple computational prediction tools: PROVEAN,^28^ FATHMM,^29^ MVP,^30^ FATHMM-MKL,^31^ LIST-S2,^32^ LRT,^33^ Mutation Assessor,^34^ SIFT,^35^ SIFT4G,^36^ PrimateAI,^37^ DANN,^38^ DEOGEN2,^39^ EIGEN PC,^40^ FATHMM-XF,^41^ and Mutation Taster.^42^ These tools were employed to assess the potential impact of the identified variants on protein structure and function.

### Protein Structure Acquisition

In this study, we employed computational methods to investigate the structural and dynamic characteristics of the CC2D1A protein for wild-type (WT) and mutants. At the beginning, the structure of the CC2D1A protein was obtained from Uniprot (Uniprot ID: Q6P1N0). The AlphaFold^43^ predicted structure, which has 951 amino acid residues (full length), was subjected to additional processing using the Protein Preparation module in the Maestro molecular modeling suite.^44^ This preprocessing involved adding missing hydrogen atoms, performing energy minimization, and defining protonation states at pH 7.4 to mimic physiological conditions, accurately.^45^ Next, a force field via OPLS3 ^46^ was used to optimize the geometry of the protein structure through constrained minimization with heavy atom convergence to a 0.3 Å root-mean-square deviation (RMSD). Further, 1 μs classical all-atom MD simulations were conducted by Desmond for the AlphaFold-predicted structure to obtain low energy conformations for nonstructured regions (the regions with low per-residue model confidence scores (pLDDT)). This prolonged simulation phase was crucial for allowing the protein, especially its nonstructured regions, to explore a wide range of conformations and settle into stable configurations.^47^ This ensures that any transiently unstable or unfolded regions have adequate time to achieve conformational stability, providing a solid foundation for more detailed analyses. Subsequently, 200 ns MD simulations were performed to relax the structure. This relaxed structure was used in clustering analysis to determine the most populated state of the protein. The analysis of the RMSD between these structures was determined to be 1.242 Å, indicating a relatively small deviation in their overall conformations. Eventually, the conformer with the lowest energy structure from the most populated cluster was selected, and mutations were introduced in this structure and used in the further analyses.

### All-Atom MD Simulations

The structure that was obtained from clustering was used in further MD simulations. The WT and the mutations (PRO192LEU, GLU518LYS, GLN506ARG, PRO532LEU, GLY781GLU, and GLY781VAL) were prepared using Schrodinger’s Maestro molecular modeling suite. (Figure S1) These structures were solvated in an orthorhombic box with the TIP3P water model.^48^ The simulation medium was neutralized using 0.15 M NaCl. OPLS3 force field is utilized for water, ions, and proteins.^45^ Nosé–Hoover thermostat was used to set temperature at 310 K,^49^ and pressure was set to 1.01325 bar and controlled with MTK barostat.^50^ 1000 frames were collected for the 100 ns simulations, and the simulations were repeated by 3 times. Three independent simulation trajectories were concatenated to obtain one single 3000-frame trajectories. Residue–residue contact frequencies were calculated with mdciao^51^ using concatenated trajectories.

### RMSD and Root Mean Square Fluctuations (RMSF) Analysis

After 300 ns (3 × 100 ns) MD simulations, we obtained RMSD, and RMSF analyses to evaluate variations in the residue positions of the CC2D1A structure. RMSD values of the position differences of backbone atoms between the mutant and WT structures were calculated throughout the MD simulations. RMSD offers a quantitative assessment of the overall structural changes, making it a crucial tool for evaluating how mutations influence the protein’s conformational stability over time.^52^ To complement the RMSD analysis and gain insights into the flexibility of specific regions within the CC2D1A protein, RMSF calculations were also conducted. The RMSF values were derived by analyzing the fluctuation of each residue’s position relative to its average position over the course of the MD simulations, providing a residue-level understanding of the protein’s dynamic properties.^52^

### SMART Analysis: Domain Characterizations

The protein domain architecture analysis was conducted using the SMART.^53^ This tool was employed to identify and characterize the structural elements and functional domains within the CC2D1A protein sequence. SMART’s integrated database and prediction algorithms enabled the detection of CC2D1A domains, along with their precise positional information within the protein sequence. In order to facilitate the understanding of CC2D1A’s modular organization and potential functional implications in cellular processes.

### Computational Prediction of Mutation Hotspots Using HotSpot Wizard 3.1

Mutation hotspots across protein variants were identified using HotSpot Wizard 3.1.^54^ This tool analyzed the protein sequences to identify functionally relevant positions. The analysis incorporated sequence conservation data, correlated mutations, protein structure stability predictions, and catalytic site annotations to identify promising mutation targets. Default parameters were used for the hotspot calculations.

### Allosteric Network Changes Analysis with Protein Structure Network (PSN)

To identify the allosteric network changes of the WT and mutants of the CC2D1A protein, PSN analyses were conducted using PSNtools and webPSN.^55,56^ The spatial arrangement of amino acids within the protein causes specific interactions that lead to conformational changes that propagate through the protein, affecting distant functional regions. To understand more deeply, the differences of the PSN in WT and the mutants were compared. In PSN analyses, we focused on CC2D1A domains, which play an important role in the interaction of CC2D1A with other proteins.

### Mutations Stability Analysis

The potential impact of six CC2D1A protein mutations on structural stability was assessed using I Mutant 2.0,^57^ a support vector machine-based tool for the prediction of protein stability changes upon single-site mutations. The Web server analyzed the difference in Gibbs free energy (ΔΔ*G*) between wild-type and mutant proteins, expressed in kcal/mol, where negative values indicate decreased stability and positive values suggest increased stability of the mutant protein compared to wild-type. All predictions were performed under standard conditions (pH 7.0 and temperature 25 °C) using the protein sequence-based version of the algorithm.

## Results

### CC2D1A’s Domain Characterizations

Based on analysis provided by SMART, the CC2D1A protein exhibits a complex domain architecture featuring three primary structural components. As shown in Figure 1, the protein contains multiple low complexity regions (LCRs) distributed throughout its entire sequence, with nine distinct segments occurring at positions 6–19, 85–109, 121–134, 201–216, 245–256, 311–345, 439–448, 475–488, and 820–834. A prominent aspect of this structure is the presence of four DM14 domains, which are repeated motifs at positions 138–195, 257–315, 349–407, and 494–552; however, their specific functions are still not well understood. The C-terminal region of the protein is marked by a single C2 domain (position 656–770), which belongs to the Protein Kinase C conserved region 2 family. This particular C2 domain is notable for its potential role in mediating calcium-dependent interactions with phospholipids, inositol polyphosphates, and other intracellular proteins, despite some C2 domains lacking calcium-binding capability. The arrangement of these domains underpins the functional diversity of CC2D1A, which includes roles in transcriptional regulation, synaptic maturation, and its involvement in the PI3K/PDK1/AKT signaling pathway.

**Figure 1:**
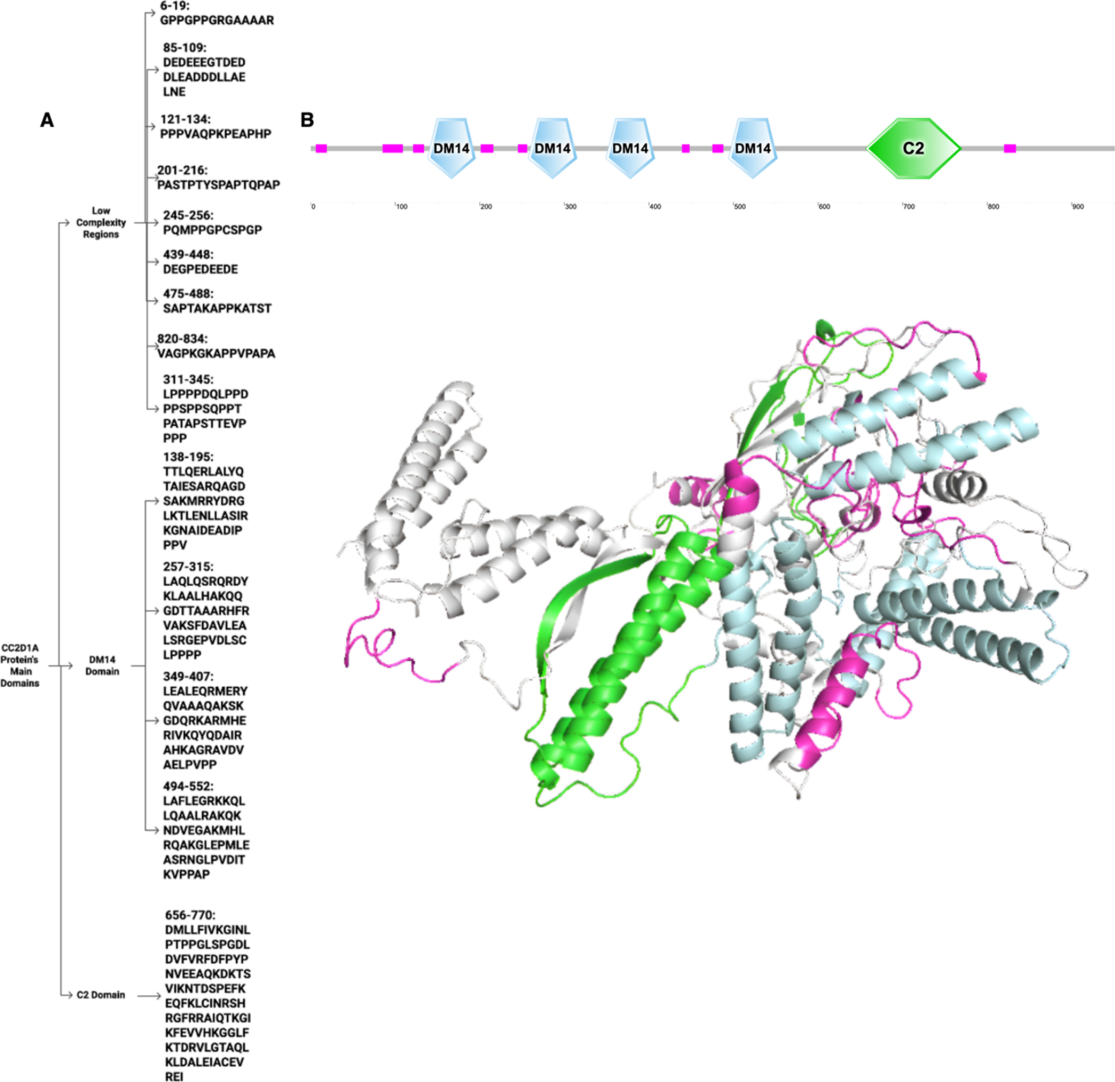
CC2D1A domains’ sequences (A). 2D and 3D CC2D1A Domains representations (B).

### Genetic Analysis of a Homozygous c.1552G > A Mutation in the *CC2D1A* in an 18-Year-Old Male with Autism and Intellectual Disability

In a regular genetic analysis at the Medical Genetics Department of Bursa Uludag University Faculty of Medicine, an intriguing case of an 18-year-old male with autism revealed a distinctive dual phenotype. The phenotype of this patient includes mental retardation, neuromotor developmental delay, epilepsy, hypertonia, hand tremors, thoracic kyphosis, synophrys, unilateral microtia, easy fatigability, and flat feet. Through whole-exome sequencing (WES), we recognized a homozygous missense mutation, c.1552G > A (GLU518LYS), within the *CC2D1A* (NM_017721.4), depicted in Figure S2. Notably, the presence of the c.1552G > A mutation in the *CC2D1A* has been associated with intellectual disability and ASD in the patient. Additionally, our investigation delved into familial genetics, offering valuable insights into the patient’s condition. Segregation analysis revealed that both parents are heterozygous carriers of the c.1552G > A mutation in the *CC2D1A*, thereby confirming its genetic underpinnings. The variant analysis results are as follows: c.1552G > A and c.1595C > T are classified as VUS (variants of uncertain significance), c.575C > T and c.2342G > A are pathogenic supporting, and c.1517A > G and c.2342G > T are classified as benign (Tables 1 and 2).

**Table 1:** Comparative Classification of Missense Variants in the *CC2D1A* across Different Genomic Databases^a^

missense variant	amino acid change	variant classifications Franklin by Genoox	variant classifications Varsome the human genomics community	variant classifications HGMD professional
c.1552G > A	GLU518LYS	VUS	Benign	not classified
c.575C > T	PRO192LEU	VUS	PM	DC
c.1517A > G	GLN506ARG	VUS	Benign	DC
c.1595C > T	PRO532LEU	VUS	PS	DC
c.2342G > T	GLY781VAL	Benign	Benign	LDC
c.2342G > A	GLY781GLU	VUS	PS	DC

a VUS: Variant of uncertain significance PS: pathogenic supporting PM: pathogenic moderate DC: disease causing mutation LDC: likely disease-causing, but with additional uncertainty.

**Table 2:** Predictive Assessment of Missense Variants in the *CC2D1A* Gene Using Various Computational Tools^a^

variants						
engines	GLU518LYS	PRO192LEU	GLN506ARG	PRO532LEU	GLY781VAL	GLY781GLU
PROVEAN	uncertain	PM	BM	PS	PM	PM
FATHMM	BM	BM	BM	BS	BM	BM
MVP	BM	BM	BM	BM	BM	BM
FATHMM-MKL	PS	PS	uncertain	uncertain	PS	PS
LIST-S2	uncertain	PS	BS	uncertain	PS	uncertain
LRT	BS	PS	BS	uncertain	uncertain	uncertain
mutation accessor	uncertain	PS	BM	uncertain	uncertain	uncertain
SIFT	uncertain	PS	BM	BS	PS	PS
SIFT4G	uncertain	PS	BM	BS	PS	PS
PrimateAI	uncertain	BS	BS	uncertain	uncertain	uncertain
BLOSUM	uncertain	uncertain	uncertain	uncertain	uncertain	uncertain
DANN	PS	uncertain	BS	uncertain	uncertain	uncertain
DEOGEN2	uncertain	uncertain	BM	uncertain	uncertain	uncertain
EIGEN PC	uncertain	uncertain	BM	uncertain	PM	PM
FATHMM-XF	uncertain	uncertain	BM	BM	PS	PS
mutationtaster	uncertain	uncertain	BM	uncertain	uncertain	uncertain

a BM: Benign moderate, BS: Benign supporting, PS: pathogenic supporting, PM: pathogenic moderate.

### Key Interactions Analysis of WT and Mutant Structures

#### WT

To assess the structural stability throughout the simulations, the RMSD of the protein backbone (bb) was computed over a duration of 100 ns, with triplicate simulations (*n* = 3), as summarized in Table 3 and visually represented in Figure 2. The WT protein exhibited RMSD values of 2.66 ± 0.51, 2.22 ± 0.28, and 2.12 ± 0.30 Å, as recorded from the repeated simulation sets (see Figure S3, upper panel, and Table S1). Collectively, the average RMSD for the WT backbone atoms remained stable at values consistently below 2.3 Å, as illustrated in Figure 2, which suggests that the system reached a conformational equilibrium with minimal deviation, indicative of a relatively stable protein structure. Additionally, a RMSF analysis was performed to evaluate the flexibility of various regions of the protein during the simulation, the results of which are depicted in Figure S3 (lower panel). The RMSF plot provides a detailed depiction of the mobility of individual residues, with higher RMSF values signifying regions with increased flexibility. The analysis indicated that all simulation trajectories reached a stable RMSD within the first 20–30 ns, reflecting an early stabilization of the overall protein conformation.

**Figure 2:**
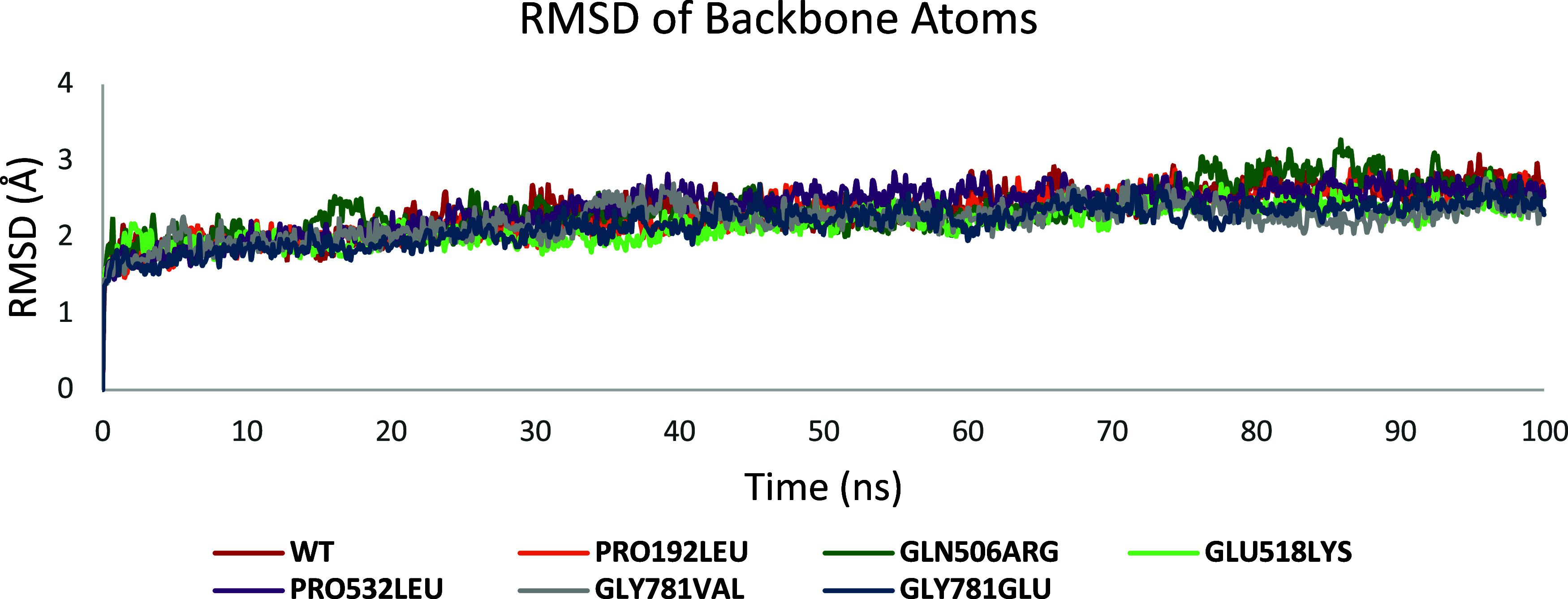
Average RMSD of backbone atoms (in Å) for WT and CC2D1A mutants over simulation time.

**Table 3:** Average and Standard Deviations (SD) of the RMSD for the WT and CC2D1A Mutations, Expressed in Å

	WT	GLU518LYS	GLN506ARG	PRO532LEU	GLY781GLU	GLY781VAL	PRO192LEU
average	2.34	2.18	2.36	2.38	2.18	2.21	2.33
SD	0.30	0.25	0.32	0.31	0.27	0.23	0.31

#### GLU518LYS

Figure 2 depicts the average RMSD across three replicate runs for the GLU518LYS mutant, illustrating how the structural deviation evolved throughout the all-atom MD simulations. The mean RMSD across these three runs was calculated to be 2.18 ± 0.25 Å, with individual values of 2.06 ± 0.23, 2.16 ± 0.34, and 2.31 ± 0.36 Å. Figure S4 (upper panel) shows the RMSD trajectories for the three replicate simulations (GLU518LYS-1, GLU518LYS-2, and GLU518LYS-3). For the initial 40 ns, all three simulations exhibited nearly identical RMSD trends. After this period, small divergences began to appear, with RMSD differences remaining relatively minor, ranging from 2 to 3 Å. Figure S4 (bottom panel) represents RMSF plots of three independent runs. When analyzing the average RMSD values of the GLU518LYS mutant compared to the WT structure, the mutant demonstrated a slightly reduced RMSD (2.18 Å for the mutant versus 2.34 Å for the WT). This subtle decrease in RMSD indicates a potential enhancement in structural stability introduced by the GLU518LYS mutation. Such a change may suggest that the substitution of glutamic acid with lysine at position 518 affects the dynamic behavior of the protein, possibly resulting in stronger intramolecular interactions or reduced flexibility, thereby stabilizing the overall protein conformation.

Neighborhood analyses were performed to explore the local environmental impacts of mutations on the protein structure. These analyses focused on identifying residues that interacted with the mutated site within a 3.5 Å radius, excluding interactions with the nearest four-bonded atoms, in both the WT and mutant forms. The aim was to identify interactions that were either disrupted or newly established as a result of the mutation and to evaluate their effects on the intraprotein interaction network. For residue 518, interactions associated with both the WT GLU518 and the mutant LYS518 were visualized in the three-dimensional structure (Figure 3). Additionally, Figure 4 provides a detailed comparison of the neighborhood interactions surrounding the GLU518 (WT) and LYS518 (mutant) residues in the CC2D1A. This analysis evaluates contact frequency, defined as the number of occurrences where a specific residue is located within a 3.5 Å radius of the reference amino acid. In Figure 4A, GLU518 is primarily observed to interact with residues THR120, LYS75, GLU119, SER118, and ALA117. The distance distribution data, illustrated in panel 4B, provides a quantitative representation of the probability of these residues being found at various distances from GLU518. The data reveal that the most frequent contacts are THR120 (99%), LYS75 (85%), GLU119 (39%), SER118 (5%), and ALA117 (2%). For the mutant residue LYS518, Figure 4C highlights that its most frequent interactions are with residues GLU119, SER118, ALA117, THR120, and ASP85. The distance distribution data in Figure 4D reveals that these interactions occur less frequently compared to those of GLU518 in the WT structure except ALA117 and SER118. Specifically, the interaction frequencies for LYS518 are GLU119 (32%), SER118 (22%), ALA117 (16%), THR120 (13%), and ASP85 (12%). A comparison of contact frequencies revealed a reduction in interactions between residue 518 and THR120 and GLU119 in the mutant, suggesting a potential decrease in stability in this region. In contrast, interactions with SER118 and ALA117 were more prominent in the mutant form, indicating possible changes in local flexibility or spatial arrangement. Notably, LYS75 was exclusively present in the WT structure, while ASP85 was uniquely observed in the mutant, highlighting mutation-induced alterations in the local interaction network. These changes may have significant implications for the protein’s structural integrity and function, warranting further investigation to understand their impact.

**Figure 3:**
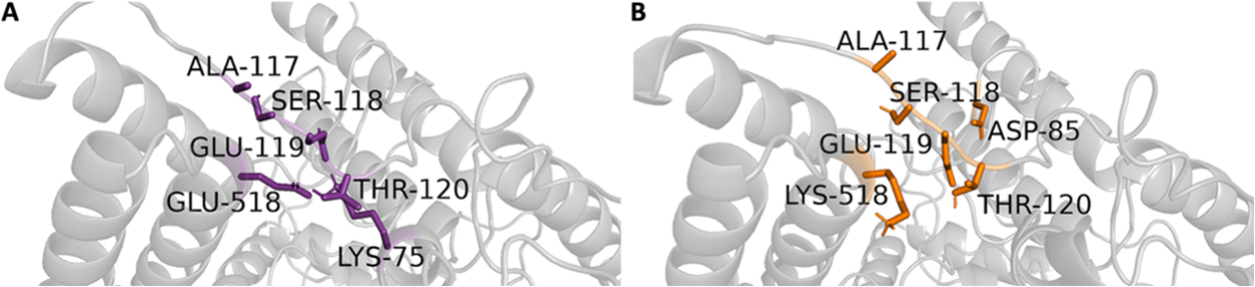
In silico neighborhood interaction analysis of (A) WT GLU518 are shown as purple sticks, (B) residues interacting with the mutant LYS518 are depicted as orange sticks.

**Figure 4:**
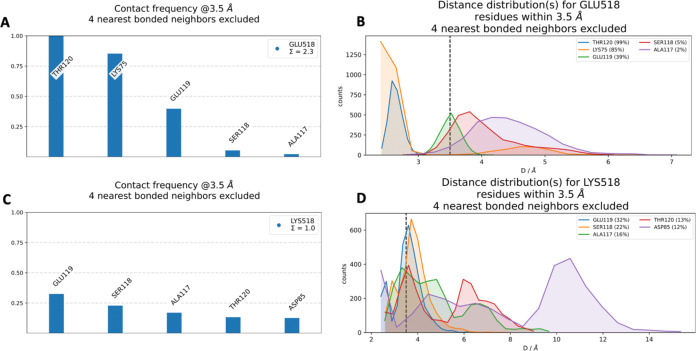
(A) Contact frequency for GLU518. (B) Distance distribution for GLU518. (C) Contact frequency for LYS518. (D) Distance distribution for LYS518.

#### GLN506ARG

The backbone atoms of the GLN506ARG mutant exhibited RMSD trajectories in the simulations, with an average RMSD value of 2.36 ± 0.32 Å (Table 3) that was recorded at 2.55 ± 0.52, 2.30 ± 0.38, and 2.23 ± 0.29 Å (Figure S5, upper panel). During the final 20 ns of the MD simulations, the first run of MD simulation for this mutant displayed slightly higher RMSD values compared to those observed in the subsequent two simulations. However, this increase in RMSD is not substantial. A consistency in RMSF values was seen throughout all iterations of the simulations, reflecting a uniform flexibility among the residues under study (Figure S5, lower panel).

The interaction residues associated with WT GLN506 and mutant ARG506 are shown in the 3D structure in Figure 5. Additionally, Figure 6 illustrates the contact frequencies of residues within a 3.5 Å radius of GLN506 and ARG506. The key interacting residues identified were ASP557 and ASN554. As shown in Figure 6B, GLN506 in the WT interacts most frequently with ASP557 (2%) and ASN554, but both interactions are relatively weak, occurring in less than 5% of the simulation time. This indicates a low interaction stability for these residues throughout the simulations. In contrast, for ARG506 in the mutant, the highest interaction frequency is observed with ASP557 (21%), followed by ASN554 (2%) and ASP556 (2%), as shown in Figure 6C,6D. The analysis indicates that ARG506 exhibits potentially stronger and more stable interactions with neighboring residues compared to GLN506. Furthermore, ARG506 establishes new contacts with ASP556, which was not observed for GLN506 in the WT. These additional interactions were found within a 3.5 Å radius of ARG506, highlighting a mutation-induced alteration in the local interaction network.

**Figure 5:**
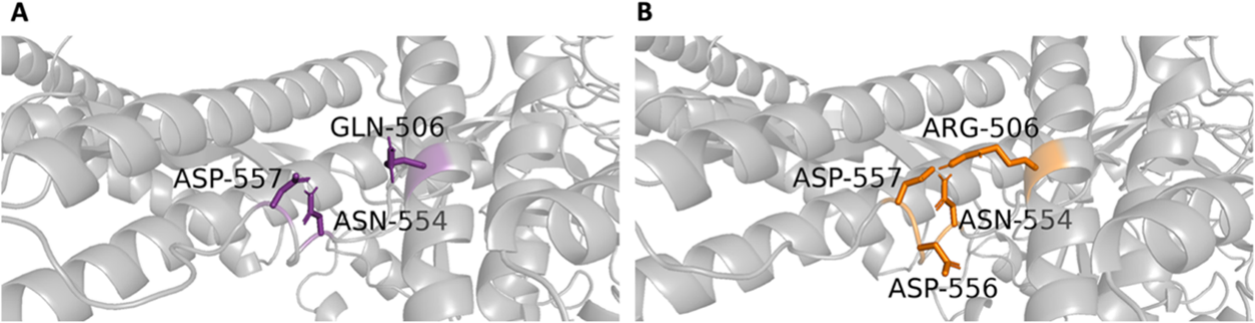
*In silico* neighborhood interaction analysis of (A) WT GLN506 are shown as purple sticks, (B) residues interacting with the mutant ARG506 are depicted as orange sticks.

**Figure 6:**
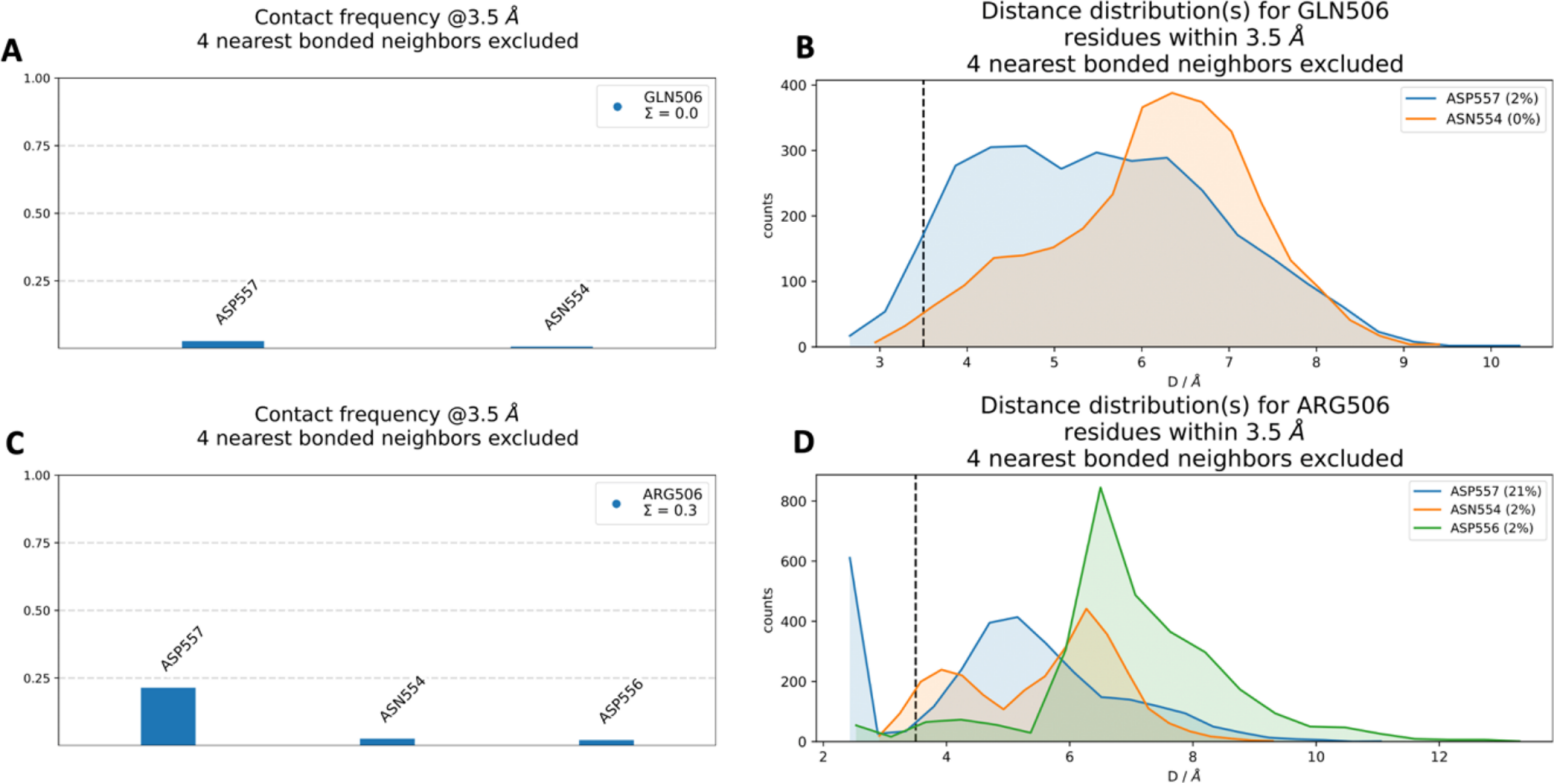
(A) Contact frequency for GLN506. (B) Distance distribution for GLN506. (C) Contact frequency for ARG506. (D) Distance distribution for ARG506.

#### PRO532LEU

The RMSD values for the backbone atoms of the PRO532LEU mutation are given in Figure S6. For all three repeats, the average RMSD is calculated as 2.38 ± 0.3 Å (Table 3). All MD simulation runs showed relatively a similar trend in RMSD values (see Figure S6, upper panel). The calculated average RMSD value for the mutant PRO532LEU compared to WT was slightly higher (2.34 ± 0.30 and 2.38 ± 0.31 Å for the WT and PRO532LEU, respectively). Figure S5 in the lower panel shows the RMSF values for the simulation repeats. Except for the residues between 20 and 50 for the first run, similar values were observed for all three repeats.

The neighborhood interactions involving WT PRO532 and mutant LEU532 are illustrated in the 3D structure presented in Figure 7. The contact frequency of interactions involving WT PRO532 and mutant LEU532 with neighboring residues within a 3.5 Å radius is presented in Figure 8. The residue with the highest contact frequency in both scenarios is THR470, as indicated by the largest bar. Figure 8B details the distance distribution, revealing that for PRO532 in the WT structure, THR470 exhibits the highest contact frequency, occurring 72% of the time. Other notable interactions include PRO471 (6%) and ARG469 (1%). For the mutant LEU532, interactions were observed with multiple residues, including THR470, ARG469, ALA465, PRO471, and PRO466. The distance distribution for LEU532 interactions reveals contact frequencies of THR470 (61%), ARG469 (10%), ALA465 (7%), and PRO471 (3%). Comparative analysis shows that the contact frequencies of THR470 and PRO471 within 3.5 Å of residue 532 decreased from the WT to the mutant. Conversely, the frequency of ARG469 interactions increased significantly for the mutant LEU532. Additionally, while ALA465 and PRO466 interactions were weaker within the 3.5 Å cutoff, they emerge as important contributors to the local interaction network when evaluated with larger cut-offs, as illustrated in Figure 8, panels C and D. This suggests that mutation-induced changes in the local interaction environment could have implications for the structural and functional dynamics of the protein.

**Figure 7:**
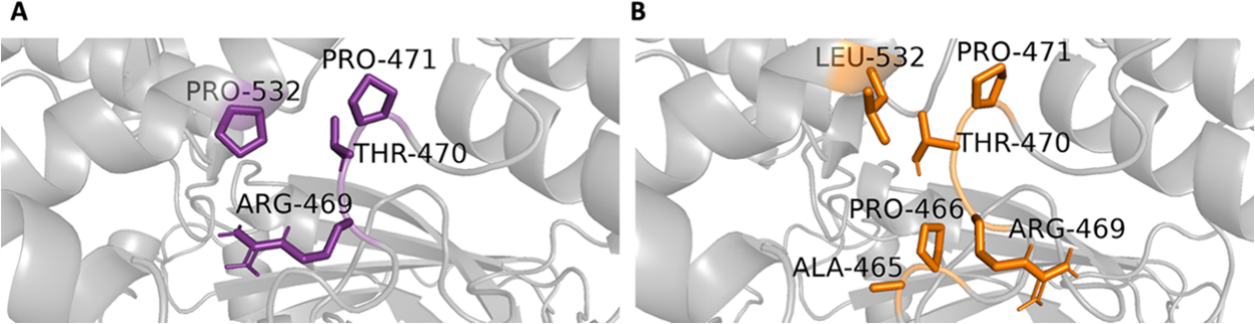
*In silico* neighborhood interaction analysis of (A) WT PRO532 are shown as purple sticks, (B) residues interacting with the mutant LEU532 are depicted as orange sticks.

**Figure 8:**
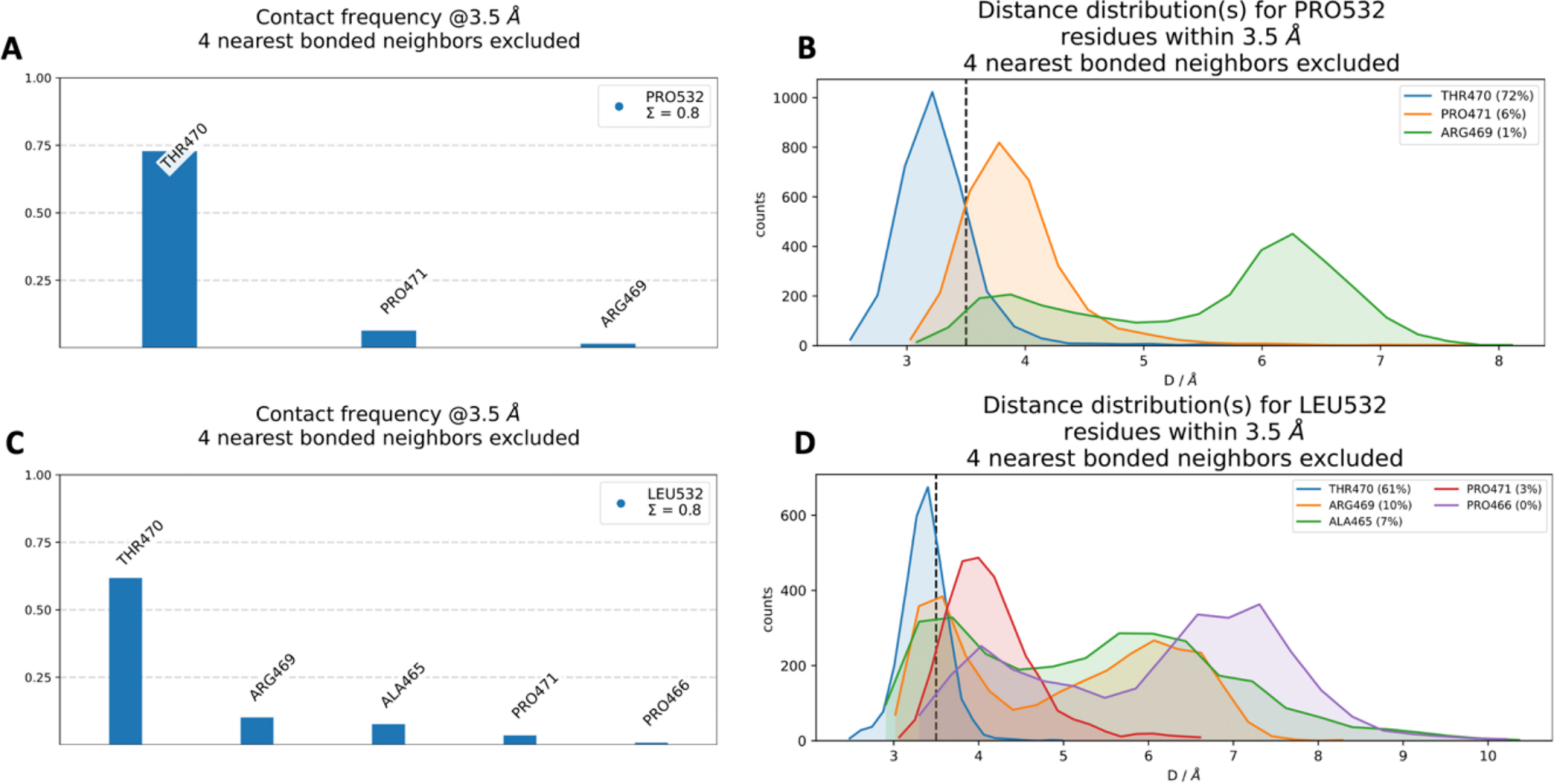
(A) Contact frequency for PRO532. (B) Distance distribution for PRO532. (C) Contact frequency for LEU532. (D) Distance distribution for LEU532.

#### GLY781GLU

The backbone RMSD plot for the GLY781GLU mutant is represented in Figure S7. Results from three independent MD simulations revealed RMSD averages of 2.29 ± 0.45, 2.21 ± 0.28, and 2.03 ± 0.25 Å, with an overall average RMSD of 2.18 ± 0.27 Å, as summarized in Table 3. The lower panel of Figure S7 represents the RMSF values for the residues. Across all simulation repeats, the RMSF values showed consistent patterns. However, in the first simulation, the RMSF values for residues between 850 and 950 deviated slightly from the average values observed in this region.

The residues involved in neighborhood interactions around WT GLY781 and mutant GLU781 are visualized in the 3D structure shown in Figure 9. The frequency of interactions between residues GLY781 and GLU781 and their neighboring residues within a 3.5 Å radius is detailed in Figure 10. Notably, SER238 emerged as the primary interaction partner in both the WT and mutant forms. Additional interactions with nearby residues, including SER238, ASN666, and SER239, were also observed. As illustrated in Figure 10B, the WT GLY781 displayed interaction frequencies with SER238 (19%), ASN666 (5%), and SER239 (1%). In contrast, the mutant GLU781 demonstrated increased interaction frequencies with a broader range of residues, including SER238, SER239, ASN666, GLN317, and ALA237. Specifically, the distance distribution analysis for GLU781 interactions revealed notable frequencies with SER238 (61%), SER239 (16%), ASN666 (10%), GLN317 (8%), and ALA237 (8%). This analysis highlights that the interaction frequency of SER238 within 3.5 Å of residue 781 increased markedly from the WT to the mutant form, suggesting that GLU781 establishes more frequent and potentially stronger interactions with its surrounding residues. Additionally, the interactions with GLN317 and ALA237 were unique to the mutant GLU781, as these residues did not form significant interactions with the WT GLY781. These findings indicate that the GLY781GLU mutation alters the local interaction network, potentially enhancing the stability and dynamics of the mutant structure.

**Figure 9:**
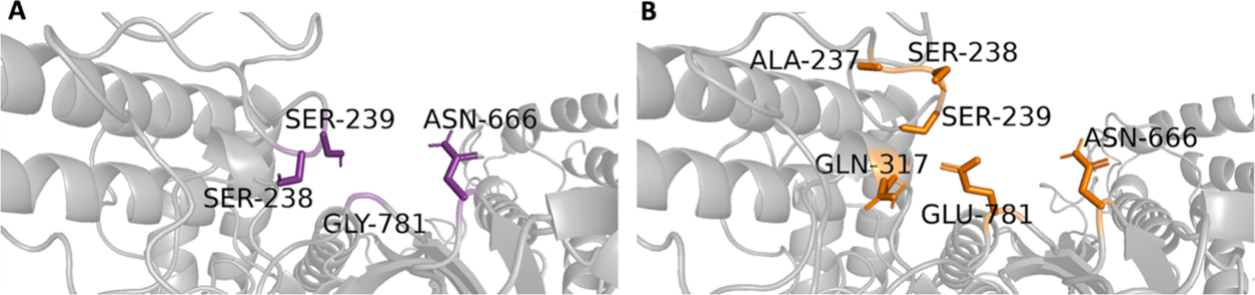
*In silico* neighborhood interaction analysis of (A) wild type GLY781 are shown as purple sticks, (B) residues interacting with the mutant GLU781 are depicted as orange sticks.

**Figure 10:**
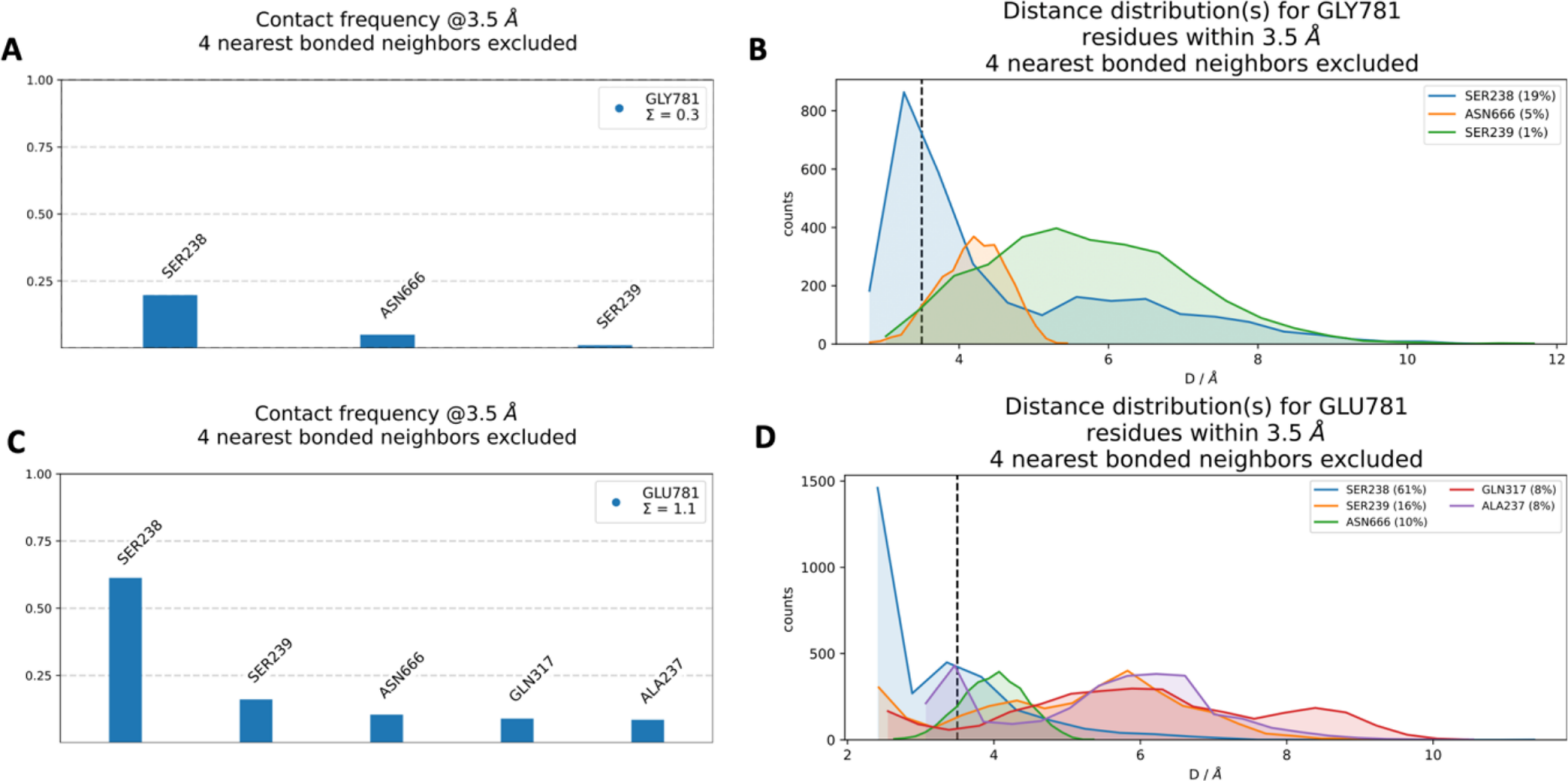
(A) Contact frequency for GLY781. (B) Distance distribution for GLY781. (C) Contact frequency for GLU781. (D) Distance distribution for GLU781.

#### GLY781VAL

The RMSD plot in the upper panel of Figure S8 depicts the simulation replicates. All three replicates exhibited similar RMSD trends for the backbone atoms. The average backbone RMSD values for the simulations were 2.15 ± 0.28, 2.30 ± 0.37, and 2.18 ± 0.26 Å, with an overall average of 2.21 ± 0.23 Å (Table 3). When comparing the average RMSD values of all WT replicates to those of the GLY781VAL mutant, the mutant showed slightly lower RMSD values, indicating that the mutation may not significantly alter the overall protein conformation. RMSF analysis revealed consistent values for most amino acid residues across the simulation replicates. However, elevated RMSF values were observed between residues 810–840 in the second simulation replicate of the GLY781VAL mutant, as shown in the lower panel of Figure S8.

The residues interacting with WT GLY781 and mutant GLU781 are illustrated in the 3D structure (Figure 11). In Figure 12B, the distribution of peak contact distances between GLY781 and SER238 reveals a contact frequency of 19%, indicating that SER238 is the predominant interaction partner for the WT residue. Additionally, GLY781 forms interactions with ASN666 (15%) and SER239 (1%). In contrast, Figure 12C demonstrates that the GLU781 mutation results in the highest contact frequencies with SER238, ASN666, SER239, and GLN317. The distance distribution analysis shows that the peak contact frequency for GLU781 with SER238 and ASN666 is 12% each, with additional contacts formed with SER239 (1%). The interaction frequency with SER238 decreases in the mutant compared to the WT, whereas the frequency of interaction with ASN666 increases in the mutant. The interaction frequency with SER239 remains consistent at 1% for both WT and mutant forms at a 3.5 Å cutoff. However, a closer examination of the distance distribution plots (Figure 12, panels B and D) reveals that SER239 demonstrates a higher interaction frequency in the WT structure when evaluated using a larger cutoff distance. These findings suggest that the GLY781GLU mutation alters the local interaction network, potentially influencing the stability and function of the protein. Such interactions likely play a pivotal role in maintaining the structural integrity and functional dynamics of the protein.

**Figure 11:**
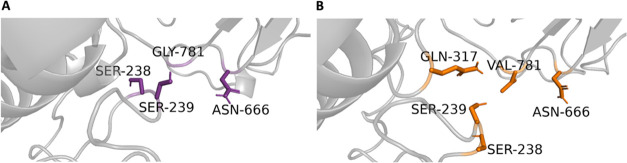
*In silico* neighborhood interaction analysis of (A) wild type GLY781 are shown as purple sticks, (B) residues interacting with the mutant VAL781 are depicted as orange sticks.

**Figure 12:**
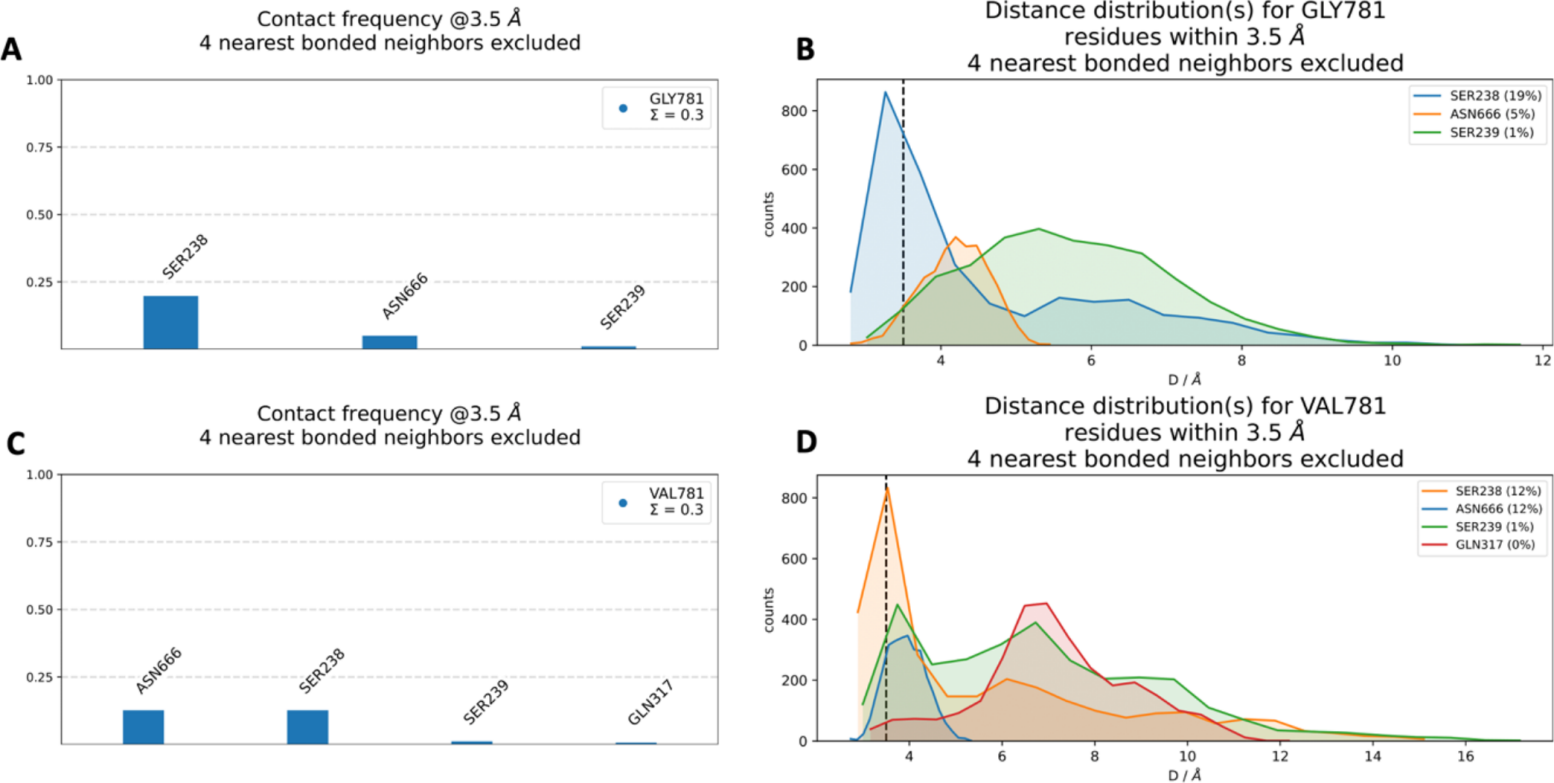
(A) Contact frequency for GLY781. (B) Distance distribution for GLY781. (C) Contact frequency for VAL781. (D) Distance distribution for VAL781.

#### PRO192LEU

The simulation repeats for the PRO192LEU mutation demonstrated consistent RMSD values of 2.23 ± 0.29, 2.19 ± 0.39, and 2.56 ± 0.42 Å (Figure S9), indicating structural stability across all replicates. A comparison of the WT RMSD average (2.34 ± 0.30 Å) with the PRO192LEU RMSD average (2.33 ± 0.31 Å) revealed nearly identical values, suggesting that the PRO192LEU mutation does not significantly impact the overall stability of the protein structure. The RMSF profiles for all three simulation repeats were generally consistent, reflecting stable residue flexibility throughout the protein.

The interactions observed in both the WT and PRO192LEU mutant are visualized in the 3D structure shown in Figure 13. In Figure 14A, the most frequent interaction for PRO192 is with ARG143, exhibiting a contact frequency of 1.00. Additional interactions include TYR147, LEU176, LEU173, and THR172. Figure 14B represents distance distribution plots for PRO192 These interactions are characterized by following frequencies: ARG143 (99%), TYR147 (32%), and LEU173 (1%). For the mutant LEU192, Figure 14C shows that ARG143 remains the primary interaction partner with a contact frequency of 1.00, alongside additional interactions with TYR147, GLY169, THR172, and LEU176. The distance distribution for LEU192 is consistent with that of PRO192, with the most frequent interaction distances occurring around 3.5 Å. These include ARG143 (100%), TYR147 (28%), GLY169 (19%), THR172 (7%), and LEU176 (4%). Overall, ARG143 is identified as the most critical residue for interactions with both PRO192 and LEU192, maintaining strong and frequent interactions, typically within a distance of 3.5 Å. Comparing the WT with the PRO192LEU mutant, the interaction with ARG143 remains equally robust in both cases. However, the interaction frequency with TYR147 slightly decreases in the mutant, while interactions with THR172 and LEU176 show modest increases. Notably, GLY169 forms interactions exclusively with the mutant LEU192, whereas LEU173 interactions are specific to the WT PRO192, with a contact frequency of 1%, likely due to the 3.5 Å cutoff used in the analysis, as shown in Figure 14B. These findings highlight subtle yet important differences in the interaction network caused by the PRO192LEU mutation, which could have implications for protein dynamics and function.

**Figure 13:**
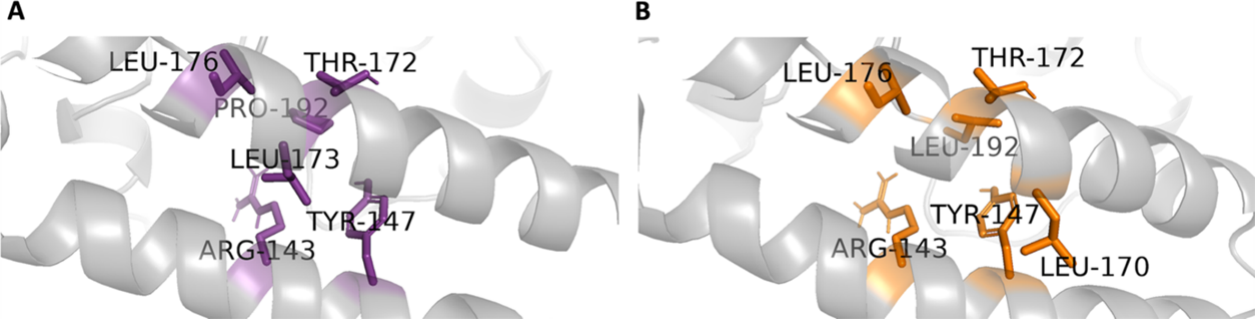
*In silico* neighborhood interaction analysis of (A) wild type PRO192 are shown as purple sticks, (B) residues interacting with the mutant LEU192 are depicted as orange sticks.

**Figure 14:**
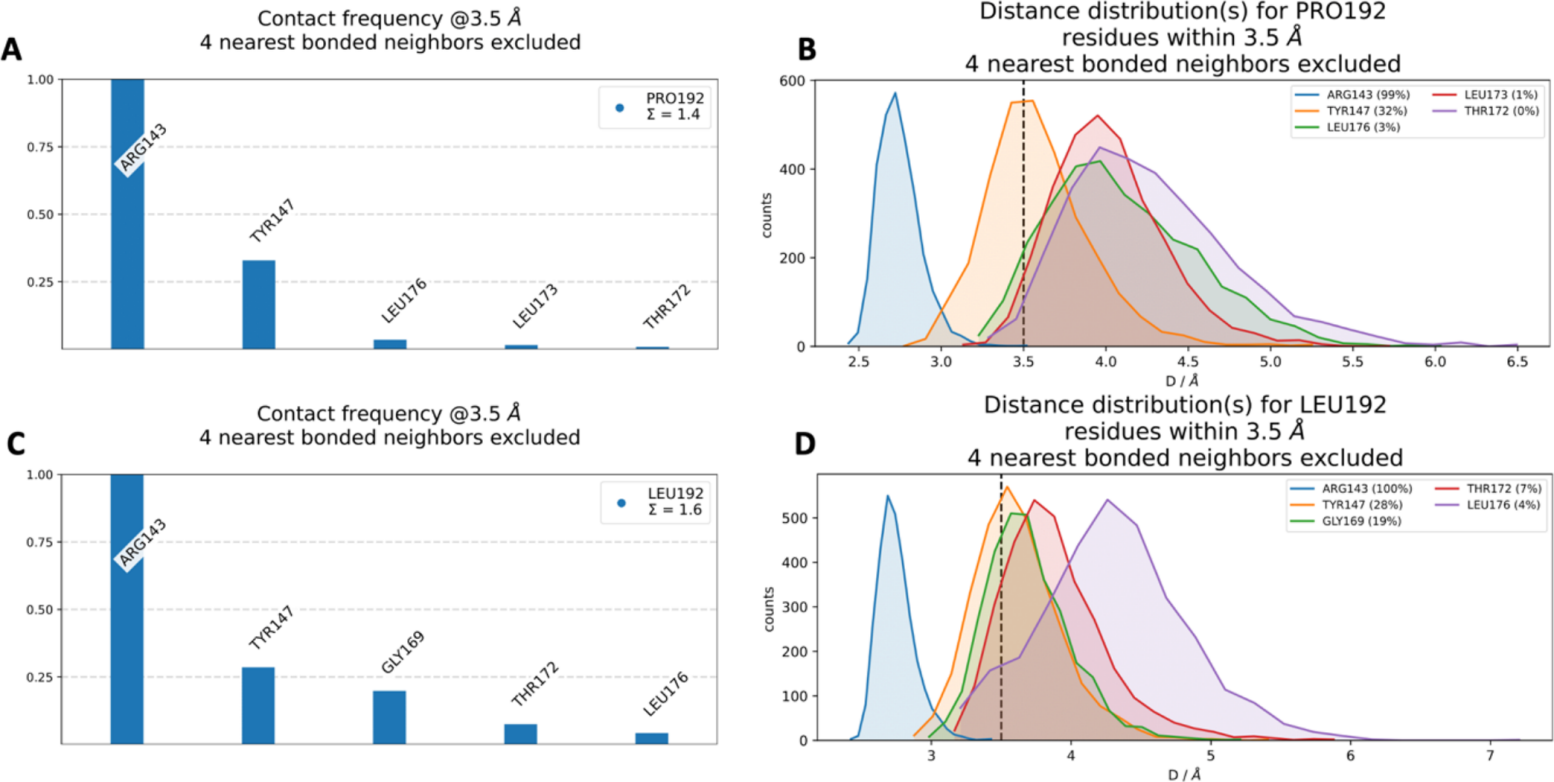
(A) Contact frequency for PRO192. (B) Distance distribution for PRO192. (C) Contact frequency for LEU192. (D) Distance distribution for LEU192.

### C2 Domain Hotspots across Protein Variants

The investigation of mutation hotspots across multiple protein variants, conducted using Hotspot Wizard,^54^ identified both conserved and unique features compared to the WT structure, as outlined in Table S8. For the C2 domain (residues 656–770), position VAL691 emerged as a highly conserved residue found in several variants, including GLU518LYS, GLN506ARG, GLY781GLU, GLY781VAL, and PRO532LEU. The WT structure exhibited key residues at GLU693, CYS717, and GLY724. The GLN506ARG variant displayed unique mutations at ARG749 and VAL750 while retaining the conserved VAL691 residue. Variants GLY781GLU and GLY781VAL shared several residues with the WT (CYS717, GLY724, GLU693, and VAL691) but showed a distinct mutation at GLN755. Similarly, the PRO532LEU variant featured specific mutations at THR669 and GLN755 while preserving VAL691. The GLU518LYS variant maintained residues at VAL691, GLU693, and ARG726, closely resembling the WT structure. Notably, GLN755 was identified as a recurring mutation site in multiple variants, including GLY781GLU, GLY781VAL, and PRO532LEU, highlighting its potential importance in protein function or stability.

### Allosteric Communication Network Variations in CC2D1A Mutants

The analysis of allosteric communication networks in the WT CC2D1A protein and its various mutants focused on the C2 domain due to its well-characterized role in mediating calcium-dependent interactions with phospholipids, inositol polyphosphates, and various intracellular proteins. Although some C2 domains lack calcium-binding functionality, they retain other critical interaction capabilities. This study identified four essential amino acid residues (LEU673, SER674, PRO675, and ASP677) on the surface of the WT C2 domain that did not actively contribute to the allosteric communication network in mutant variants, as depicted in Figure 15. To further investigate the roles of these residues, single-point mutations replacing the native amino acids with Alanine were performed (I Mutant analyses). The results, presented in Figure 15, demonstrated how these mutations influenced protein stability, offering valuable insights into the contribution of these residues to the domain’s overall structural framework. The findings suggest that while these residues may not directly participate in the communication network, they play a critical role in maintaining the domain’s characteristic molecular interactions and structural robustness. This underscores their potential importance in ensuring the domain’s ability to mediate key functional processes.

**Figure 15:**
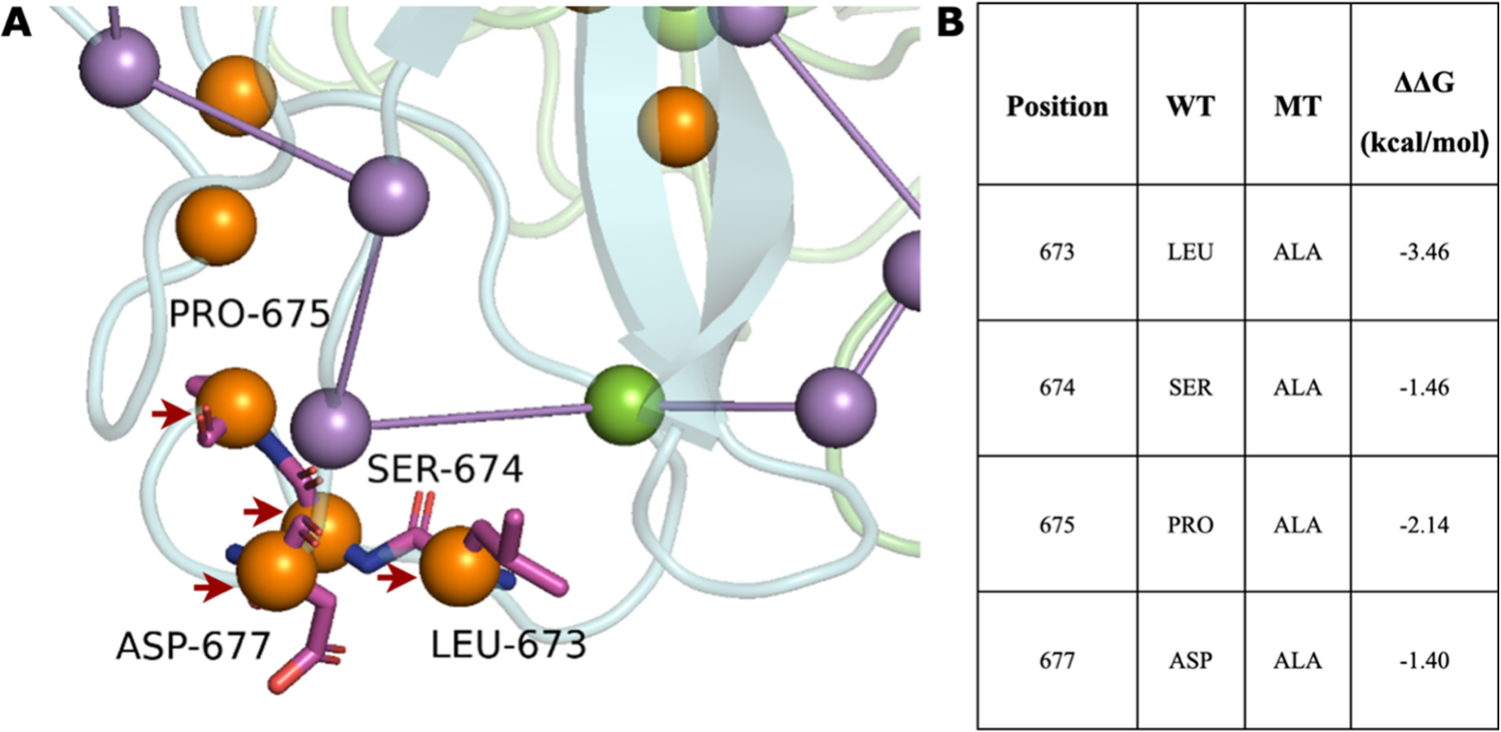
Four key amino acids (LEU673, SER674, PRO675, and ASP677) in the C2 domain’s wild-type configuration (orange) are absent in mutant allosteric pathways (purple).

Figure 16 illustrates the distinct signaling pathways observed in both WT and GLU518LYS mutant proteins. Analysis of several mutations revealed varying effects on protein stability and allosteric communication. Alanine mutations led to protein destabilization (ΔΔ*G* = −0.52 kcal/mol), while GLN506ARG showed modified allosteric communication and reduced stability (ΔΔ*G* = −0.09 kcal/mol). PRO532LEU displayed a network pattern similar to GLU518LYS, with absent amino acid residues likely disrupting protein–protein interactions (ΔΔ*G* = −0.05 kcal/mol). GLY781GLU generated a unique network predominantly outside the C2 domain and enhanced protein stability (ΔΔ*G* = +0.13 kcal/mol), while GLY781VAL decreased protein stability (ΔΔ*G* = −0.31 kcal/mol). PRO192LEU caused the most severe destabilization (ΔΔ*G* = −0.80 kcal/mol) by completely disrupting the normal allosteric network.

**Figure 16:**
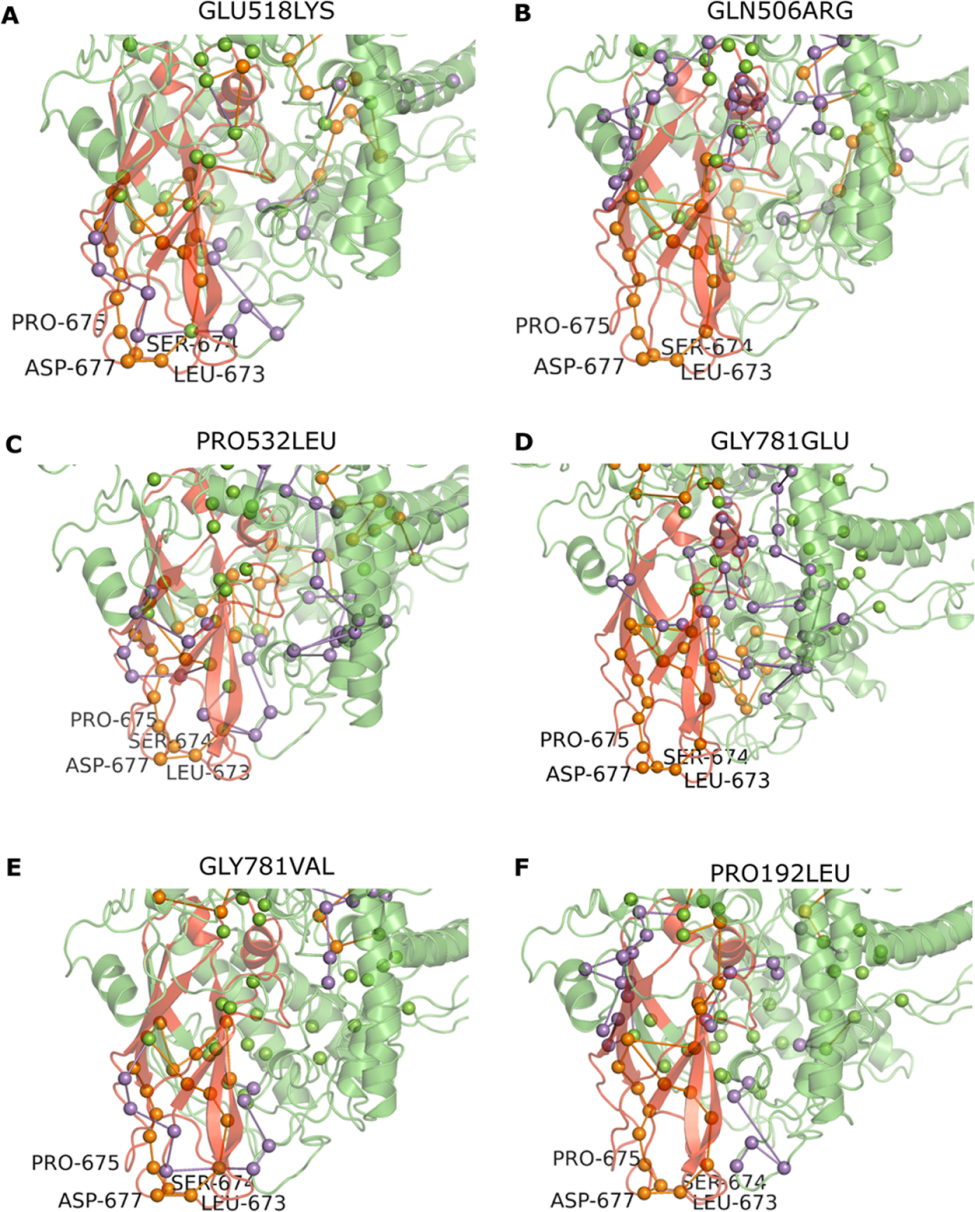
Allosteric communication network variations in CC2D1A mutants.

A notable finding across all mutants was the consistent absence of four key amino acids (LEU673, SER674, PRO675, and ASP677) from the C2 domain network. This suggests impaired protein–protein interactions and potential functional changes in the CC2D1A protein. Our *in silico* mutant analyses demonstrated that mutations at these specific positions generally reduced protein stability, as evidenced by predominantly negative ΔΔ*G* values.

## Discussion

The *CC2D1A* plays a pivotal role in a wide array of neurological and developmental disorders, highlighting its critical importance in human biology. These conditions include ASD, intellectual disability, seizures, autosomal recessive nonsyndromic intellectual disability, renal dysplasia, heterotaxy, and ciliary dysfunction.^2,17,18^ Such a broad spectrum of associated disorders underscores the *CC2D1A*’s essential role in developmental processes and its contribution to cellular and molecular pathways integral to neurodevelopment and organogenesis. Its functional versatility suggests that disruptions in *CC2D1A* can have cascading effects on multiple systems, thereby contributing to the pathogenesis of these disorders. To unravel the molecular underpinnings of *CC2D1A*-associated disorders, it is crucial to examine how genetic mutations in this gene affect its structural and functional integrity. Mutations in *CC2D1A* may destabilize its protein structure, disrupt critical interaction networks, or impair signaling pathways, ultimately leading to the manifestation of disease phenotypes. However, a comprehensive understanding of these mutations and their effects on protein function has remained elusive.

In our study, WES has uncovered a c.1552G > A (GLU518LYS) missense mutation within the *CC2D1A* of an 18-year-old male, linking this specific genetic alteration to his diagnosed intellectual disability and autism. Missense mutations within the exons of the *CC2D1A*, c.575C > T (PRO192LEU), c.1517A > G (GLN506ARG), c.1595C > T (PRO532LEU), c.2342G > T (GLY781VAL), and c.2342G > A (GLY781GLU) have been implicated in causing heterotaxy and ciliary dysfunction through studies using a zebrafish model.^18^ While there is a significant similarity between the human *CC2D1A* and its zebrafish equivalent, *CC2D1A*, in terms of genetic conservation, the implications of these mutations have not been established in human cell cultures or models derived from patients.^18^ Moreover, the impact of these mutations on the structure and function of the human CC2D1A protein remains poorly understood. For example, it is unclear how these mutations might affect protein stability, interaction dynamics, or domain functionality, which are critical determinants of CC2D1A’s role in signaling pathways and developmental regulation. Therefore, our study goes beyond analyzing the GLU518LYS mutation in isolation. Instead, it aims to investigate the broader implications of *CC2D1A* mutations, leveraging advanced computational biology techniques to elucidate their effects on protein conformation and interactions. By deepening our understanding of these mutations, this study seeks to shed light on the genetic and molecular foundations of complex developmental and neurological conditions, paving the way for targeted therapeutic interventions.^58–61^

MD simulations play a critical role in elucidating the structural and dynamical alterations in WT and single-point mutant proteins, offering atomic-level insights that are challenging to obtain through experimental techniques.^62–64^ By modeling protein motions over time, MD captures the conformational flexibility and dynamic interactions that govern protein function. Single-point mutations can induce significant changes in both local and global stability, flexibility, or folding by perturbing key molecular interactions such as hydrogen bonds, salt bridges, and hydrophobic contacts. Furthermore, MD simulations provide a detailed understanding of how mutations impact functional regions, such as active or allosteric sites, potentially altering binding affinity, catalytic activity, or regulatory mechanisms. Comparative analysis of WT and mutant proteins through MD facilitates the detection of subtle conformational changes, offering critical insights into disease-related mechanisms, aiding drug discovery efforts, and predicting the functional consequences of genetic variations. Thus, the findings from our study provide valuable insights into the structural and functional consequences of missense mutations in the *CC2D1A*, which is implicated in various neurodevelopmental disorders, including ASD and intellectual disability. By employing all-atom MD simulations and neighborhood interaction analyses, we have elucidated the distinct impacts of the PRO192LEU, GLN506ARG, PRO532LEU, GLY781VAL, GLY781GLUand GLU518LYS mutations on the CC2D1A protein structure and function.

### Structural Stability and Conformational Integrity

Our RMSD analysis revealed that certain mutations, specifically GLU518LYS, GLY781VAL, and GLY781GLU, did not significantly alter the overall protein structure when compared to the WT. The RMSD values for these mutants remained close to those of the WT, suggesting that these mutations might preserve the protein’s global conformational integrity. However, to more accurately reflect the implications of our findings, it is important to note that the observed RMSD values alone cannot fully rule out subtle functional effects or localized disruptions caused by these mutations. This finding underscores that not all mutations in the *CC2D1A* gene lead to drastic structural changes. These mutations may still contribute to the disease phenotype through mechanisms independent of global structural destabilization, potentially involving subtle alterations in protein interactions, localized conformational changes, or disruptions in signaling pathways. For example, the slight deviations observed in the GLY781VAL and GLY781GLU mutants suggest that while the overall structure is maintained, localized changes could influence protein function. Conversely, the PRO192LEU, GLN506ARG, and PRO532LEU mutations showed higher RMSD values, indicating potential disruptions in protein stability. The PRO192LEU mutation, in particular, exhibited pronounced fluctuations in specific regions of the protein, as indicated by both RMSD and RMSF analyses. These findings suggest localized instabilities that may significantly impact the protein’s functional capabilities. Taken together, these observations highlight that while some mutations maintain global structural integrity, their effects on protein dynamics and function may still be profound, necessitating further investigation into their impact on molecular interactions and signaling pathways.

### Neighborhood Interaction Analysis and Functional Implications

The neighborhood interaction analysis provided a detailed understanding of how these mutations alter local interaction networks within the CC2D1A protein. For instance, the GLU518LYS mutation led to the emergence of a new interaction with ASP85 while losing the interaction with LYS75 observed in the WT. This alteration suggests a reorganization of the local interaction network at the mutation site, potentially reshaping the structural and functional landscape. Similarly, the GLY781VAL and GLY781GLU mutations introduced novel interactions with GLN317 and ALA237, interactions absent in the WT. While these mutations maintained overall structural stability, the changes in interaction patterns indicate a shift in local environments, which may influence specific functional aspects critical to CC2D1A’s role in cellular processes. The introduction of new interaction partners in the mutant forms, coupled with the loss of original partners, highlights the potential for these mutations to subtly, yet significantly, alter protein function. The PRO192LEU mutation, which resulted in a loss of interaction with LEU173 and the formation of a new interaction with GLY169, underscores the potential for mutations to reorganize local interaction networks in a manner that might disrupt normal protein function. This reorganization could be particularly relevant in the context of neurodevelopmental disorders, where precise protein interactions are crucial for normal cellular processes.^13,65–68^ CC2D1A is also essential for NF-κB signaling homeostasis, acting as a key modulator of neuron survival with dual roles in promoting neuroprotection or contributing to neurodegeneration depending on the context.^8,10,69^ Thus, alterations of CC2D1A’s interaction networks may destabilize this pathway, potentially affecting neuronal differentiation and the formation of functional neural circuits. Therefore, altered interactions in key domains of the protein could impair its role in facilitating proper cellular signaling, leading to downstream effects on neuronal development and connectivity. These findings highlight the importance of maintaining intact local interaction networks within CC2D1A to preserve its structural and functional integrity. Disruptions to these networks, even subtle ones, may have far-reaching consequences, particularly in highly sensitive systems like the nervous system, where precise molecular interactions are essential for cellular homeostasis and function. Additional studies, including experimental validation, are warranted to explore the full extent of these mutations’ effects on protein behavior and cellular processes.

### Mutational Impact on Structure and Communication Networks in the CC2D1A C2 Domain

The integrated analysis of allosteric communication networks and mutational hotspots in the C2 domain (residues 656–770) of CC2D1A revealed complex structure–function relationships. The C2 domain, recognized for its role in calcium-dependent interactions with phospholipids, inositol polyphosphates, and various intracellular proteins,^70^ demonstrated distinct patterns of network perturbation across different variants. Notably, four critical residues (LEU673, SER674, PRO675, and ASP677) identified in the WT C2 domain surface were consistently absent from the communication networks of all mutant variants, suggesting their fundamental role in maintaining domain functionality. On the other hand, the hotspot analysis provided complementary insights, with WT protein exhibiting conserved hotspots including ARG726, GLY724, CYS717, GLU693, and VAL691 within the C2 domain. Variants GLY781VAL, GLY781GLU, and GLU518LYS maintained some substantial conservation of these C2 domain hotspots, suggesting preservation of key structural elements despite the loss of the four critical allosteric network residues. This apparent contradiction between conserved hotspots and disrupted network residues implies complex allosteric mechanisms governing domain function.

In contrast, variants PRO532LEU, PRO192LEU, and GLN506PRO showed marked alterations in both hotspot distribution and allosteric networks within the C2 domain. The complete absence of C2 domain hotspots in PRO192LEU and GLN506PRO, coupled with the disruption of the four key network residues, suggests significant perturbation of domain architecture and potential impairment of characteristic molecular interactions. The recurrent appearance of GLN755 as a hotspot in multiple variants near the C2 domain boundary, combined with the l mutant analyses of network residues, indicates a complex interplay between structural stability and allosteric communication that may be critical for domain function. This integrated analysis suggests that while certain mutations preserve local structural elements as evidenced by conserved hotspots, they may still significantly impact long-range allosteric communication networks essential for domain function. These findings highlight the subtle yet crucial balance between structural conservation and dynamic communication patterns in maintaining C2 domain functionality.

### Limitations of Computational Approaches in the Study of *CC2D1A* Mutations

The analysis primarily relied on computational methods, MD simulations, and neighborhood interaction analyses, mainly without experimental validation through in vitro binding assays or functional studies in cellular models, which would be necessary to confirm the predicted effects of these mutations on protein function. While the study provided valuable insights into structural changes and protein interactions, it did not directly examine how these mutations affect CC2D1A’s role in specific cellular pathways, particularly those involved in neurodevelopment, such as Notch signaling, BMP signaling, and NF-κB pathways.

### Broader Implications and Future Directions

The findings from this study contribute to our understanding of the molecular mechanisms by which missense mutations in the *CC2D1A* gene may contribute to neurodevelopmental disorders. The fact that some mutations maintain overall structural integrity while others induce local instabilities or alter interaction networks suggests a spectrum of potential impacts on protein function. These insights underscore the importance of considering both global and local structural changes when evaluating the pathogenicity of genetic mutations. Moving forward, it would be valuable to complement these computational findings with experimental studies, such as in vitro binding assays or functional studies in cellular models, to validate the predicted effects of these mutations on protein function. Additionally, exploring the impact of these mutations on the CC2D1A protein’s role in specific cellular pathways, particularly those involved in neurodevelopment, could provide deeper insights into the pathogenic mechanisms underlying ASD and related disorders.

Overall, this study highlights the nuanced effects of missense mutations on the CC2D1A protein, offering a foundation for future research aimed at developing targeted therapeutic strategies. Mutations such as GLU518LYS, GLY781VAL, and GLY781GLU preserve structural integrity, suggesting they may contribute to disease through altered interactions or signaling pathways rather than destabilization. In contrast, PRO192LEU, GLN506ARG, and PRO532LEU mutations lead decreases in local interactions, likely impairing protein function and associated pathways. Thus, this study serves as a foundation for future research aimed at developing targeted therapies for *CC2D1A*-associated disorders. Understanding the molecular consequences of specific mutations could enable the identification of potential druggable sites within CC2D1A or its interaction partners. Additionally, these findings could inform precision medicine approaches by linking specific mutations to clinical phenotypes and tailoring interventions accordingly. By improving our understanding of how these mutations influence protein structure and function, we can better grasp the molecular underpinnings of the associated neurodevelopmental disorders and potentially identify new avenues for intervention.

## Conclusions

This study provides comprehensive insights into the structural and functional consequences of missense mutations in the *CC2D1A*, particularly focusing on their implications for neurodevelopmental disorders such as ASD and intellectual disability. Through a combination of MD simulations and neighborhood interaction analyses, we have highlighted the distinct effects of the GLU518LYS, PRO192LEU, GLN506ARG, PRO532LEU, GLY781VAL, and GLY781GLU mutations on the CC2D1A protein. Our findings demonstrate that certain mutations, such as GLU518LYS, GLY781VAL, and GLY781GLU, do not significantly alter the overall structural integrity of the protein, as indicated by their stable RMSD values. These mutations appear to preserve the global conformation of the protein, which may suggest a retained functional. This highlights the possibility that these mutations might contribute to the disease phenotype through mechanisms other than structural destabilization, potentially involving subtle changes in protein interactions or signaling pathways. In contrast, other mutations, including PRO192LEU, GLN506ARG, and PRO532LEU, exhibit relatively higher RMSD values, suggesting potential decrease in protein stability. The local instabilities and reconfigurations in interaction networks observed for these mutants indicate that they might significantly affect the protein’s function, potentially leading to altered signaling or cellular processes that contribute to the pathogenesis of neurodevelopmental disorders.

The neighborhood interaction analyses provided further insights into how these mutations impact the local interaction landscapes within the protein. Specifically, the introduction of new interactions and the loss of original ones in the mutant forms underscore the potential for these mutations to alter the functional dynamics of the CC2D1A protein. Such alterations may potentially affect CC2D1A’s involvement in cellular pathways, including NF-κB,^10^ dopamine D2 receptor,^11^ regulation of serotonin 1A^12,68,69^ and BMP signaling,^13^ and Notch signaling,^14^ all of which play essential roles in neurodevelopment. Therefore, mutations that disrupt these interactions may potentially lead to downstream effects on synaptic development and maintenance. However, additional functional studies integrating analyses are warranted to validate these findings and uncover potential therapeutic targets for treating these conditions.

Overall, this study advances our understanding of the molecular mechanisms underlying *CC2D1A*-related disorders, emphasizing the importance of both global and local structural analyses in evaluating the pathogenicity of genetic mutations. The insights gained from this work not only enhance our knowledge of disease pathogenesis but also pave the way for future research aimed at developing targeted therapeutic interventions to mitigate the impact of *CC2D1A* mutations on human health.

## Supplementary Material



## Data Availability

The simulation data for this study is available in the Zenodo repository at 10.5281/zenodo.11163230.

## Notes

Comprehensive structural analyses of the CC2D1A protein system, comparative structural analyses between wild-type CC2D1A and its mutant variants, a visualization of the c.1235T > C mutation, and extensive MD simulations data presented through RMSD and RMSF plots; the analyses cover multiple protein variants, including GLU518LYS, GLN506ARG, PRO532LEU, GLY781GLU, GLY781VAL, and PRO192LEU mutations; tabulated data presents RMSD averages and standard deviations (in Å) from multiple MD simulations replicas of both WT and mutant proteins; comparison of mutation hotspots across protein variants, with specific attention to low-complexity regions, DM14 domains, and C2 domains, providing a thorough structural context for the findings (PDF)
