# Supplementary material for: Computational Analysis of CC2D1A Missense Mutations: Insight into Protein Structure and Interaction Dynamics
Source: ACS Chem Neurosci. 2025 Jan 10;16(19):3665–81. doi: 10.1021/acschemneuro.4c00570 (PMC12498394; doi:10.1021/acschemneuro.4c00570)
Supplement: Supplementary file 1 [file cn4c00570_si_001.pdf]

SUPPORTING INFORMATION

Computational Analysis of *CC2D1A* Missense Mutations: Insight into Protein Structure and Interaction Dynamics

Anwar Abuelrub[1,2,3], Ismail Erol[1,4], Nurdeniz Nalbant Bingol[5], Sebnem Ozemri Sag[6], Sehime G. Temel[5,6,7,*], Serdar Durdağı[1,2,8,*]

[1]Laboratory for Innovative Drugs (Lab4IND), Computational Drug Design Center (HITMER), Bahçeşehir University, 34734, İstanbul, Türkiye; [2]Computational Biology and Molecular Simulations Laboratory, Department of Biophysics, School of Medicine, Bahçeşehir University, 34734, Istanbul, Türkiye; [3]Graduate School of Natural and Applied Sciences, Artificial Intelligence Program, Bahçeşehir University, 34734, Istanbul, Turkey; [4]Department of Analytical Chemistry, School of Pharmacy, Bahçeşehir University, 34351, İstanbul, Türkiye; [5]Department of Translational Medicine, Institute of Health Sciences, Bursa Uludag University, 16059, Bursa, Türkiye; [6]Department of Medical Genetics, Faculty of Medicine, Bursa Uludag University, 16059, Bursa, Türkiye; [7]Department of Histology and Embryology, Faculty of Medicine, Bursa Uludag University, 16059, Bursa, Türkiye; [8]Molecular Therapy Laboratory, Department of Pharmaceutical Chemistry, School of Pharmacy, Bahçeşehir University, 34351, İstanbul, Türkiye

Supplementary Figures

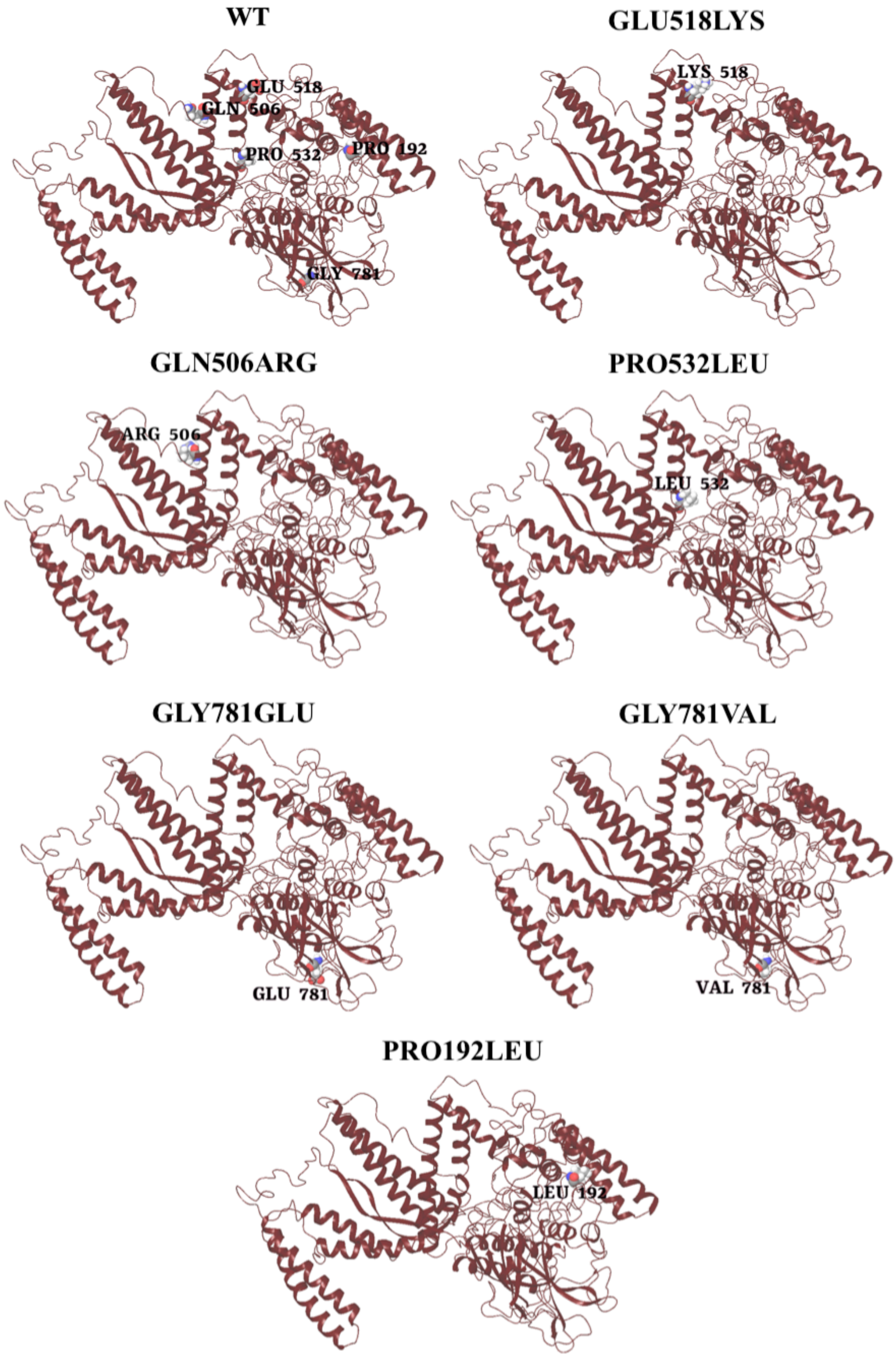

Figure S1. Structural Comparison of Wild-Type CC2D1A Protein with Mutants.

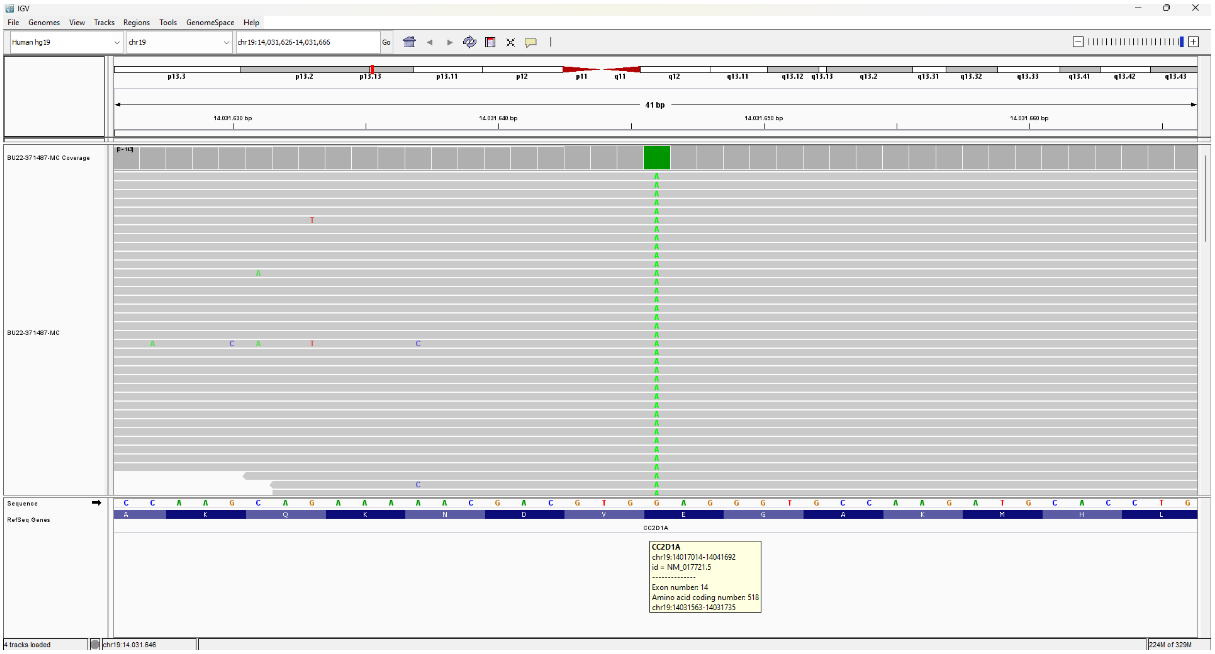

Figure S2. Integrative genomic view of c.1235T>C (p. GLU518LYS) homozygous change in CC2D1A in exon 14.

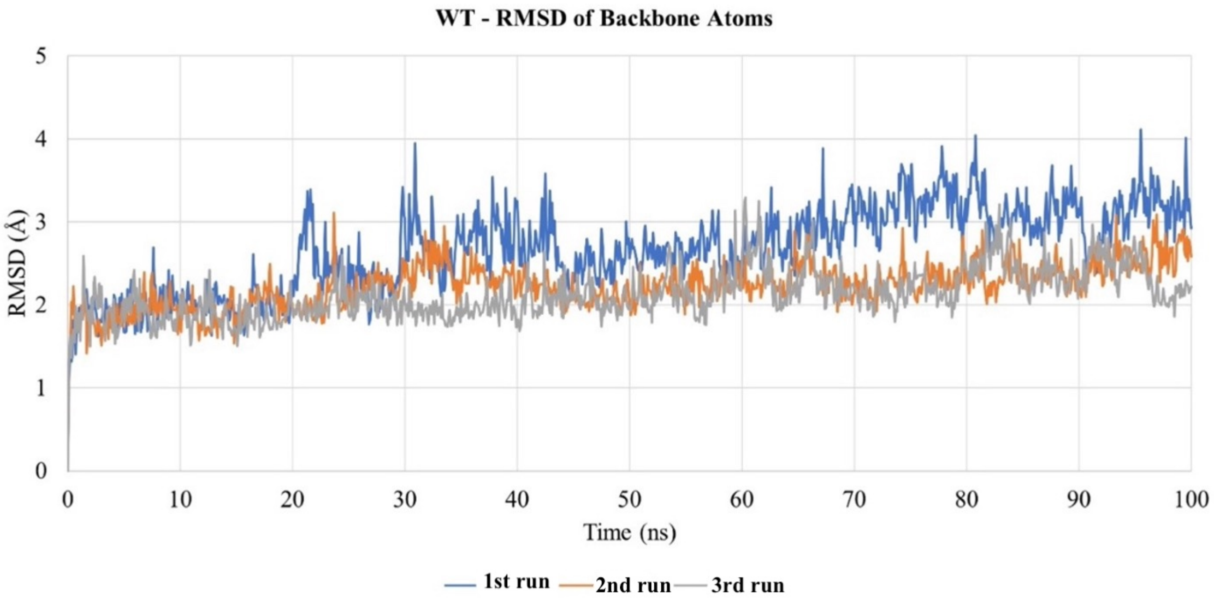

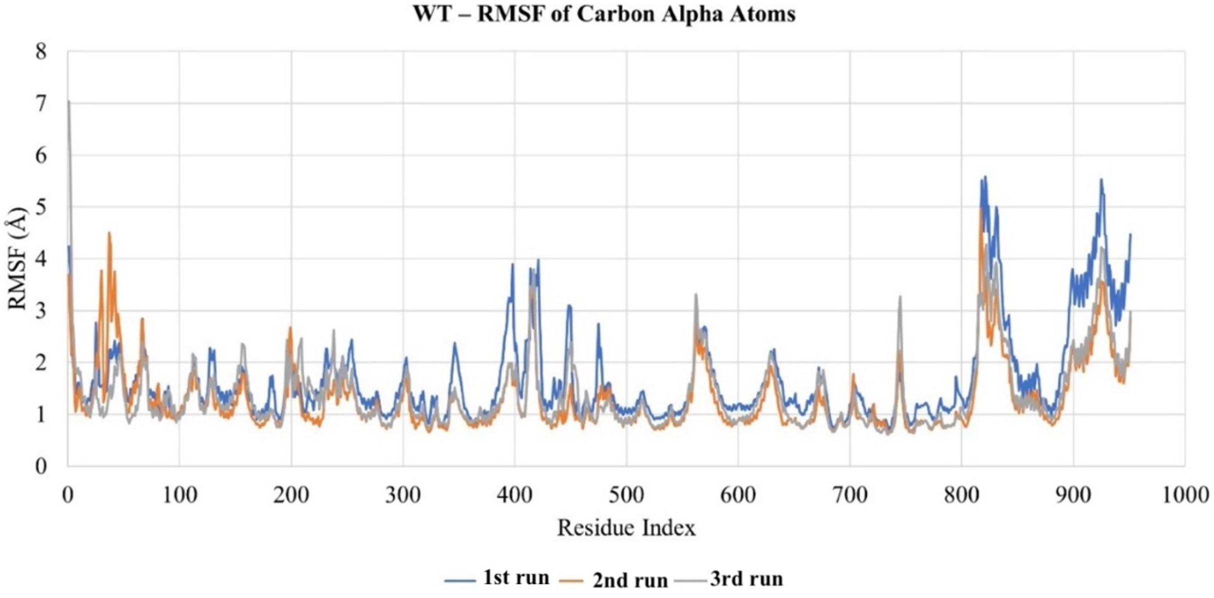

Figure S3. RMSD plots for the WT simulation replicas (upper panel), RMSF plots for the WT simulation replicas (lower panel).

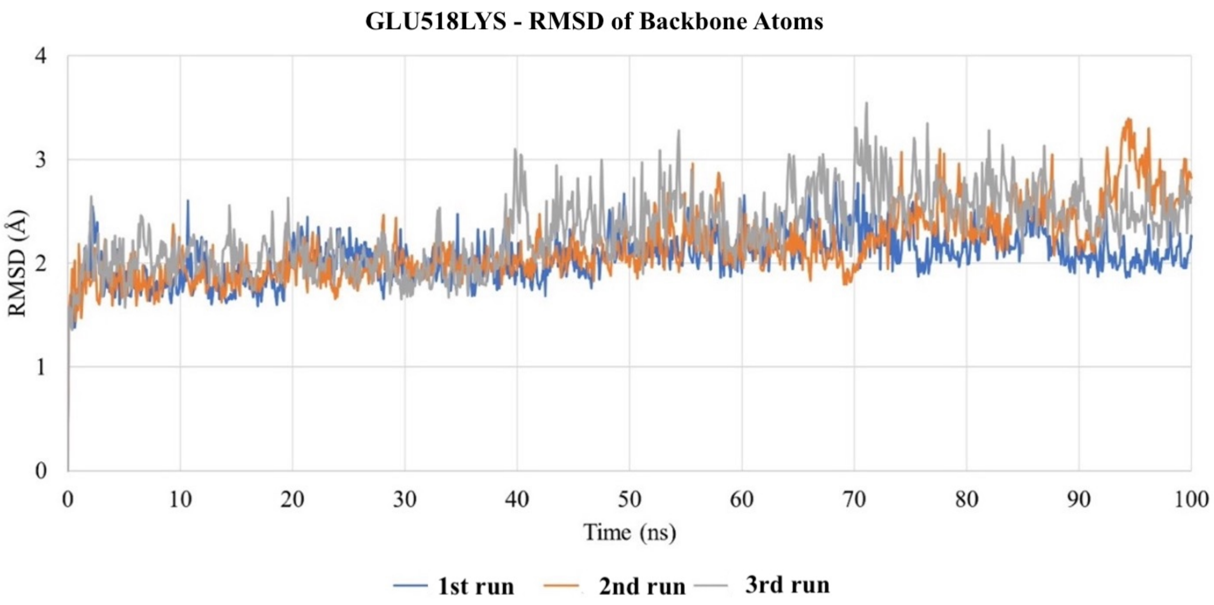

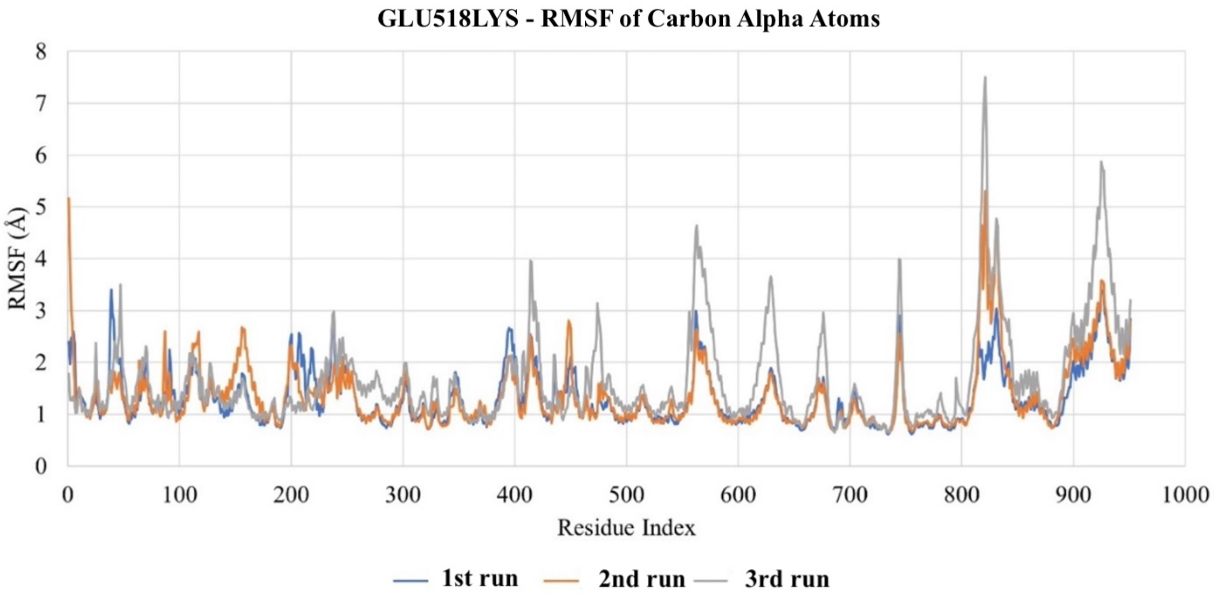

Figure S4. RMSD plots for the GLU518LYS simulation replicas (upper panel), RMSF plots for the GLU518LYS simulation replicas (lower panel).

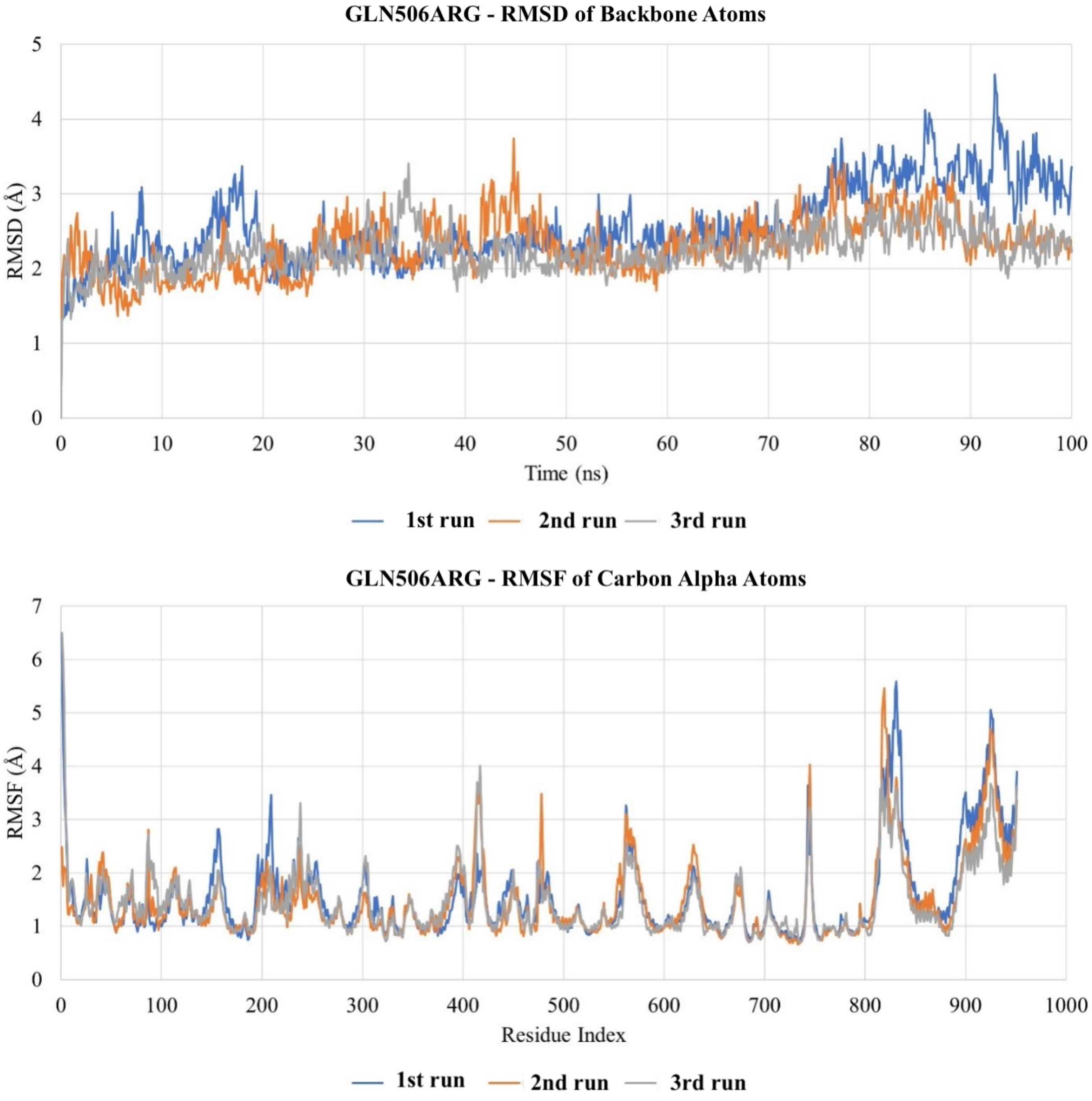

Figure S5. RMSD plots for the GLN506ARG simulation replicas (upper panel), RMSF plots for the GLN506ARG simulation replicas (lower panel).

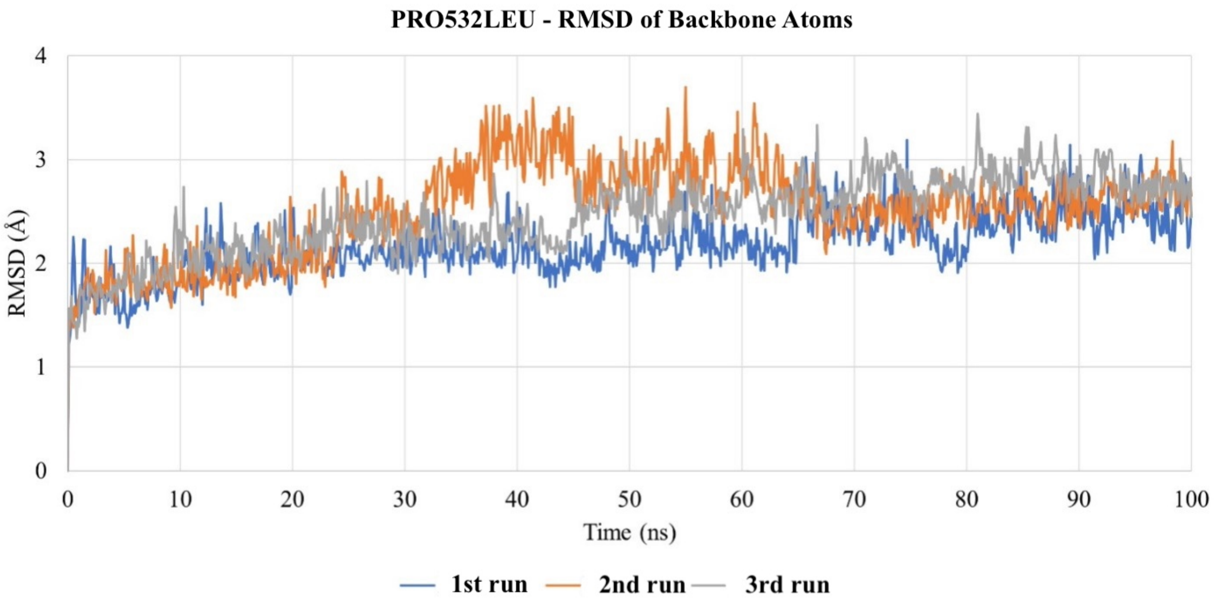

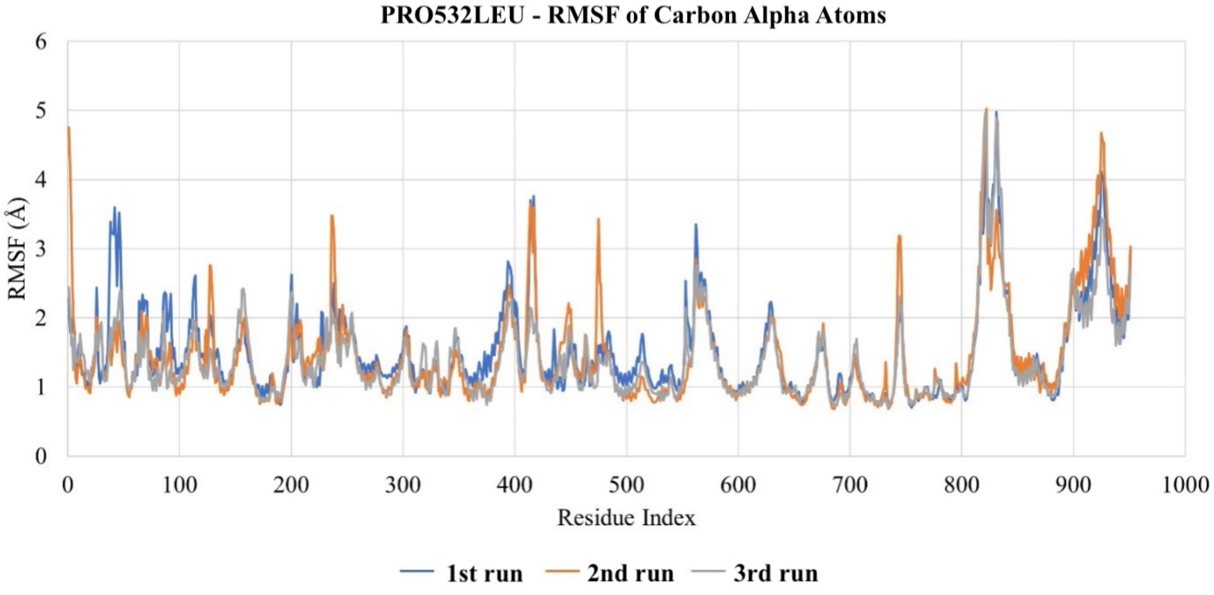

Figure S6. RMSD plots for the PRO532LEU simulation replicas (upper panel), RMSF plots for the PRO532LEU simulation replicas (lower panel).

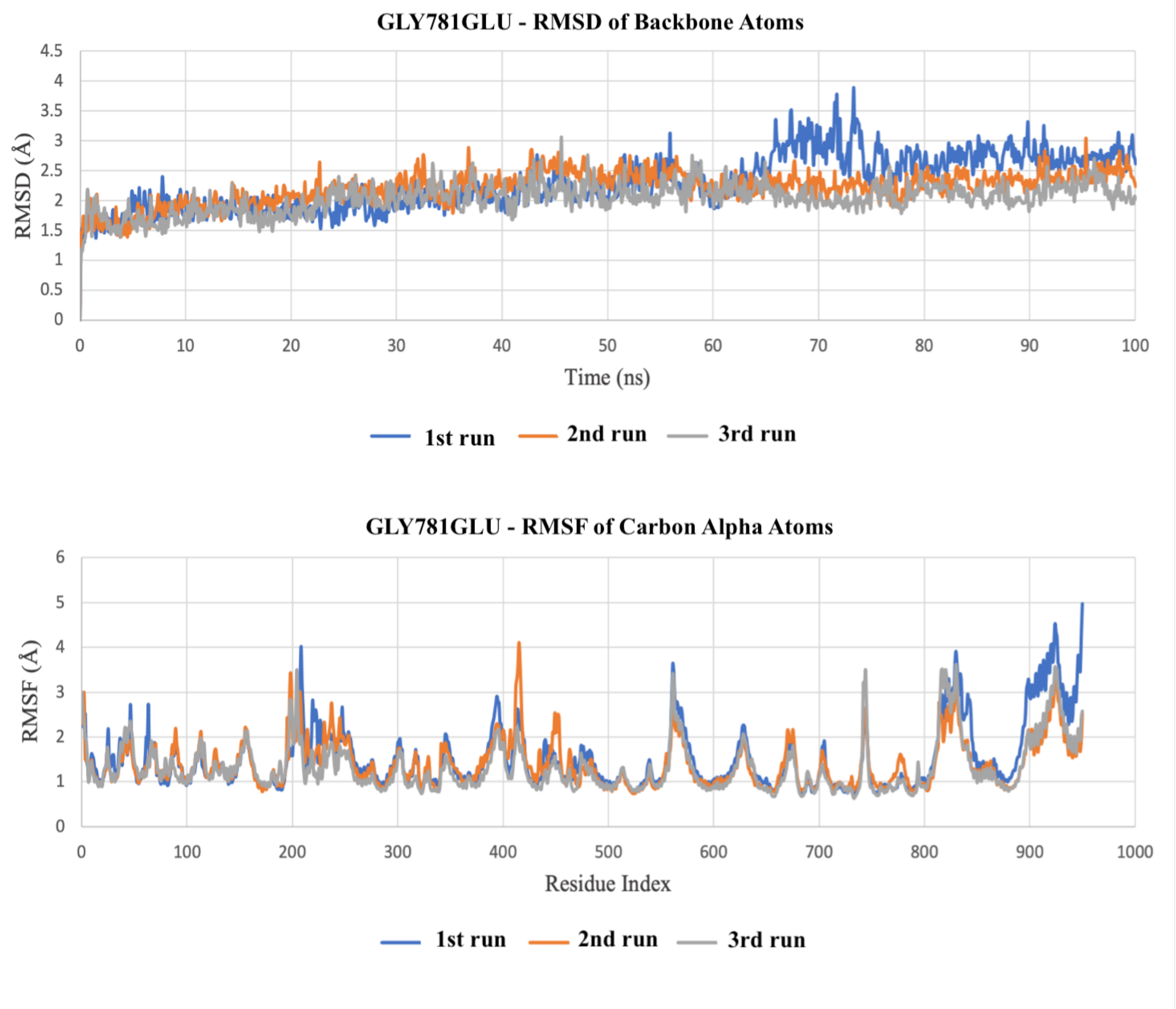

Figure S7. RMSD plots for the GLY781GLU simulation replicas (upper panel), RMSF plots for the GLY781GLU simulation replicas (lower panel).

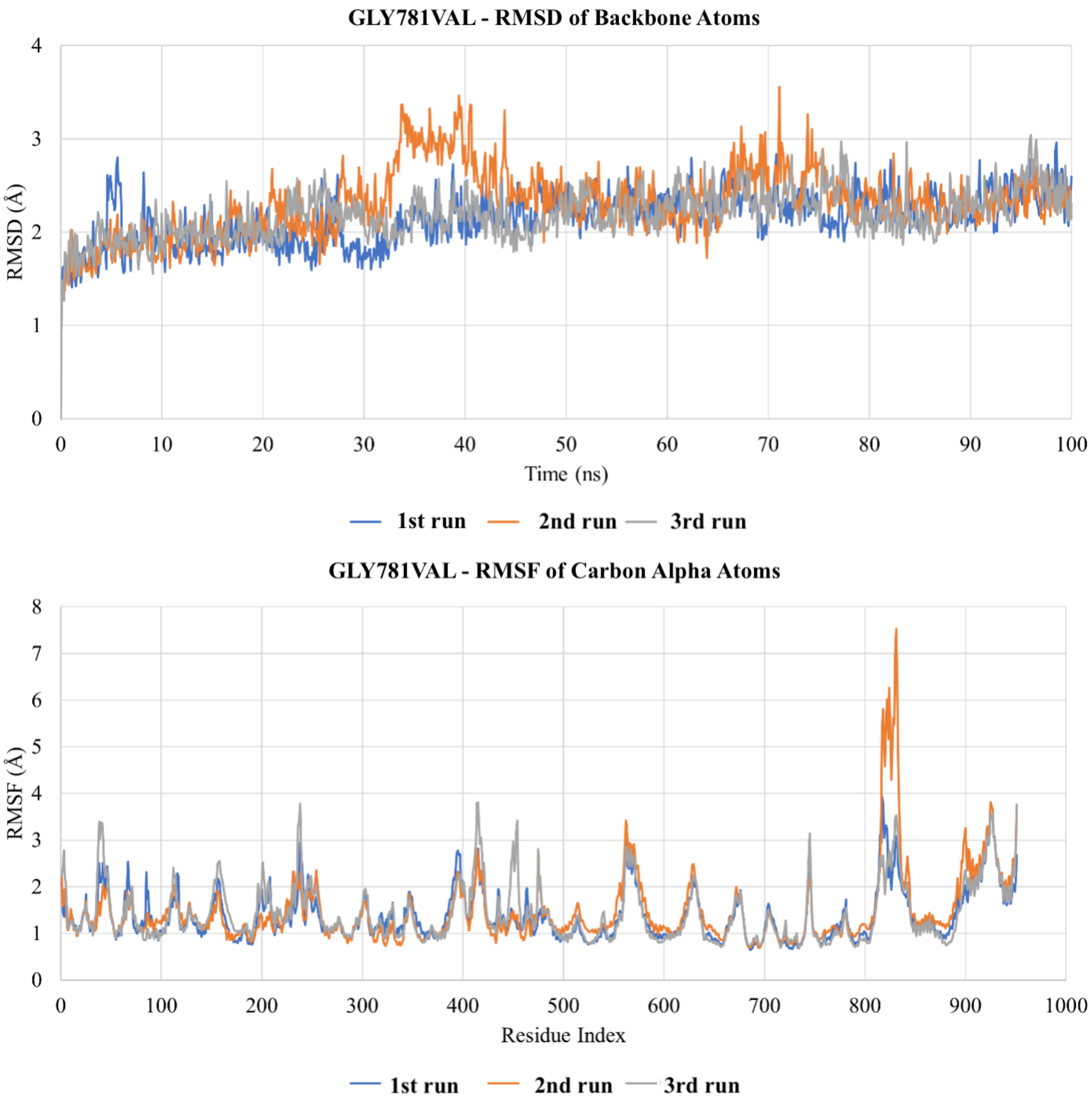

Figure S8. RMSD plots for the GLY781VAL simulation replicas (upper panel), RMSF plots for the GLY781VAL simulation replicas (lower panel).

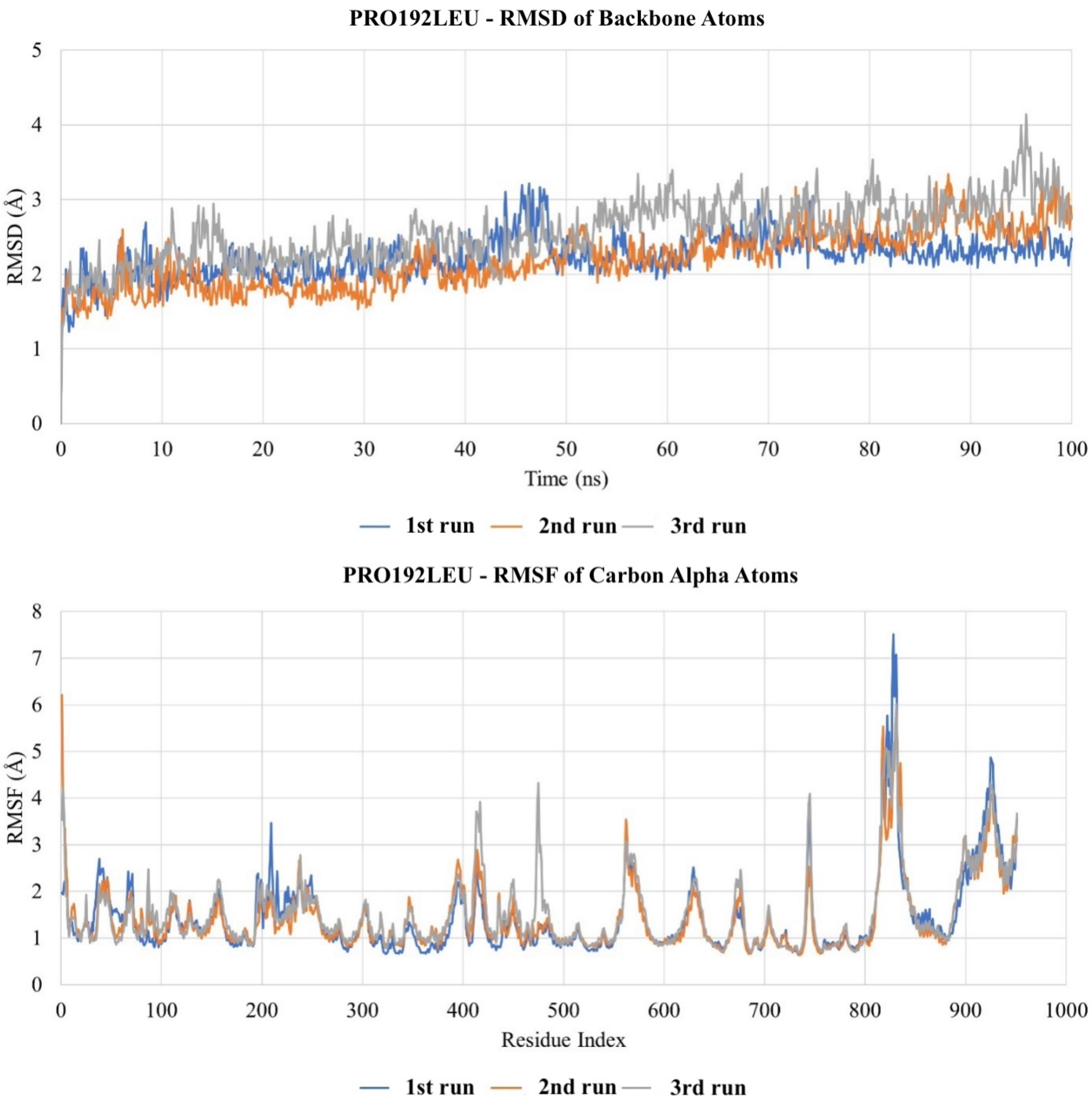

Figure S9. RMSD plots for the PRO192LEU simulation replicas (upper panel), RMSF plots for the PRO192LEU simulation replicas (lower panel).

Supplementary Tables

Table S1. RMSD averages and standard deviations in Å for WT MD replicas.

	WT-1	WT-2	WT-3	WT-A
Average	2.66	2.22	2.12	2.34
SD	0.51	0.28	0.30	0.30

Table S2. RMSD averages and standard deviations in Å for GLU518LYS MD replicas.

	GLU518LYS-1	GLU518LYS-2	GLU518LYS-3	GLU518LYS-A
Average	2.06	2.16	2.31	2.18
SD	0.23	0.34	0.36	0.25

Table S3. RMSD averages and standard deviations in Å for GLN506ARG MD replicas.

	GLN506ARG-1	GLN506ARG-2	GLN506ARG-3	GLN506ARG-A
Average	2.55	2.30	2.23	2.36
SD	0.52	0.38	0.29	0.32

Table S4. RMSD averages and standard deviations in Å for PRO532LEU MD replicas.

	PRO532LEU-1	PRO532LEU-2	PRO532LEU-3	PRO532LEU-A
Average	2.18	2.51	2.46	2.38
SD	0.31	0.44	0.38	0.31

Table S5. RMSD averages and standard deviations in Å for GLY781GLU MD replicas.

	GLY781GLU-1	GLY781GLU -2	GLY781GLU -3	GLY781GLU -A
Average	2.29	2.21	2.03	2.18
SD	0.45	0.28	0.25	0.27

Table S6. RMSD averages and standard deviations in Å for GLY781VAL MD replicas.

	GLY781VAL-1	GLY781VAL-2	GLY781VAL-3	GLY781VAL-A
Average	2.15	2.30	2.18	2.21
SD	0.28	0.37	0.26	0.23

Table S7. RMSD averages and standard deviations in Å for PRO192LEU MD replicas.

	PRO192LEU-1	PRO192LEU-2	PRO192LEU-3	PRO192LEU-A
Average	2.23	2.19	2.56	2.33
SD	0.29	0.39	0.42	0.31

Table S8. Comparative Analysis of Mutation Hotspots Across Protein Variants.

Domain	Position	WT	PRO192LEU	GLU518LYS	GLN506ARG	GLY781GLU	GLY781VAL	PRO532LEU
**Low Complexity Regions**	**6-134**			136			136	
**DM14**	**138-195**						139	
		167		167		167	167	
		175		175		175	175	
		179		179				
				184				
				185				
						189	189	
**Low Complexity Regions**	**201-256**	197		197		197	197	
		198		198			198	
		199						
				200				
		201		201			201	
		202		202				
		203						
		204		204			204	
		205		205	205			
				206	206			
			250					
			251					
			256					
**DM14**	**257-315**		259					
			262					
			265					
					281			
			282		282			
			285		285			285
					292			
**Low Complexity Regions**	**311-345**		310		310		310	
								323
		330						
		331						
**DM14**	**349-407**			361				
				364				
		365		365				
		368		368			368	
		369		369		369	369	
		372		372				
**C2**	**656-770**							669
		691		691	691	691	691	
		693		693		693	693	
							697	
		717				717	717	
							721	
		724		724		724	724	
		726		726				
					749			
					750			
			755		755		755	755
					771		771	771
			779		779		779	779
